# Algae drive convergent bacterial community assembly at low dilution frequency

**DOI:** 10.1016/j.isci.2023.106879

**Published:** 2023-05-16

**Authors:** Kaumudi H. Prabhakara, Seppe Kuehn

**Affiliations:** 1 Center for Physics of Evolving Systems, University of Chicago, Chicago, IL 60637, USA; 2 Department of Ecology and Evolution, University of Chicago, Chicago, IL 60637, USA

**Keywords:** Biological sciences, Microbiology, Soil biology

## Abstract

Microbial community assembly is a complex dynamical process that determines community structure and function. The interdependence of inter-species interactions and nutrient availability presents a challenge for understanding community assembly. We sought to understand how external nutrient supply rate modulated interactions to affect the assembly process. A statistical decomposition of taxonomic structures of bacterial communities assembled with and without algae and at varying dilution frequencies allowed the separation of the effects of biotic (presence of algae) and abiotic (dilution frequency) factors on community assembly. For infrequent dilutions, the algae strongly impact community assembly, driving initially diverse bacterial consortia to converge to a common structure. Analyzing sequencing data revealed that this convergence is largely mediated by a decline in the relative abundance of specific taxa in the presence of algae. This study shows that complex phototroph-heterotroph communities can be powerful model systems for understanding assembly processes relevant to the global ecosystem functioning.

## Highlights

• Studied assembly of algae-bacteria communities
• Algal influence on the bacterial community is strong at low dilution frequencies
• At strong algal influence, distinct bacterial communities taxonomically converge
• Convergence occurs mainly due to decrease in relative abundance of certain taxa

## Graphical abstract

**Figure:**
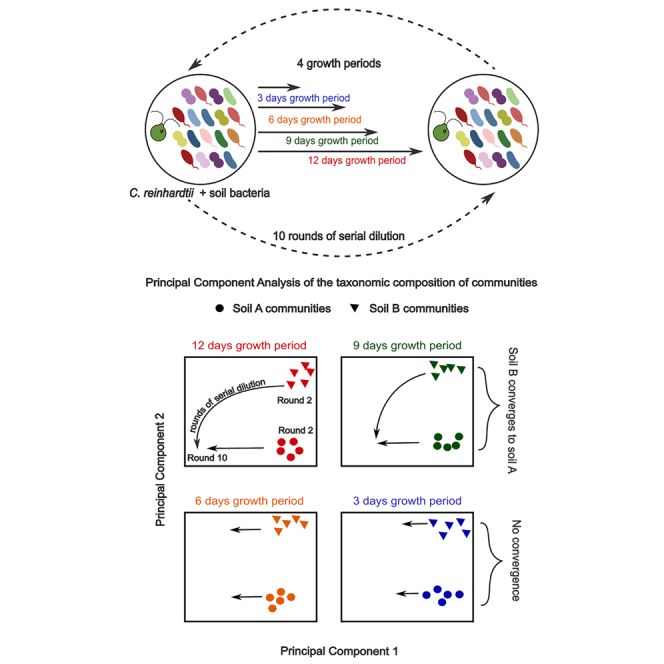

## Introduction

Building a predictive understanding of the assembly of communities is a central problem in microbial ecology. The assembly process dictates the structure of communities which ultimately impacts their stability and functional properties such as carbon cycling and primary productivity.^4^^,^^15^^,^^27^ Community assembly depends on an interplay between species-species interactions,^19^ immigration,^46^ emigration, and abiotic factors^43^ that modulate the growth and physiology of organisms in a collective. Disentangling the relative impacts of biotic and abiotic factors on the structure of a community is challenging because of the interdependence between these factors and the difficulty of controlling them independently in wild settings.

Recent work using microbial consortia has revealed that the local environment has a strong impact on the assembled community. For example, the assembly of marine bacterial communities on polysaccharide particles shows a conserved structure defined by the metabolic traits of the community members.^29^ Similarly, sequencing studies of natural communities in the ocean show that the presumed metabolic traits of the strains present are correlated with local abiotic factors such as light and nutrient levels.^43^ Further, human gut bacterial consortia display a conserved successional process of specific bacterial taxa.^8^ Collectively, these studies point to the idea that the local environment is a strong determinant of the composition of microbial consortia.

What remains unclear is how to differentiate the contributions of abiotic factors such as nutrient levels, temperature, and light intensity from the contributions of biotic factors such as species-species interactions. One context where biotic and abiotic factors can both play an important role is the assembly of communities comprised photosynthetic microbes (algae, cyanobacteria, diatoms) in association with non-photosynthetic (heterotrophic) bacteria. Phototroph-heterotroph communities are pervasive in soils and aquatic environments, where these two metabolic strategies are often found in tight symbioses.^11^^,^^17^^,^^55^^,^^60^^,^^63^ Phototroph-heterotroph symbioses typically arise from phototrophs fixing CO_2_ and excreting organic compounds^3^^,^^5^^,^^10^^,^^18^^,^^48^^,^^64^ that can be subsequently utilized by heterotrophs. However, this is not the only source of organic carbon for heterotrophic bacteria, because they can also utilize nutrients from decaying organic matter. In this sense, the assembly of bacterial communities in the presence of phototrophs is influenced by biotic interactions with the phototrophs and the abiotic availability of nutrients. In fact, studies of wild communities have shown that dissolved organic carbon supplied from terrestrial sources (allochthonous) or excreted from autotrophs (autochthonous) can impact the bacterial community taxonomically,^34^^,^^37^ functionally,^68^ and dynamically.^49^ These studies point to the idea that environmental variables, such as the availability of nutrients, influence assembly by modulating the relative importance of nutrient exchanges. However, in wild communities, causality is challenging to establish, and interactions between taxa hard to interrogate.

To overcome these challenges, we used model communities comprised a dominant phototroph interacting with naturally derived heterotrophic bacterial communities. We develop the model soil-dwelling alga *Chlamydomonas reinhardtii*, and complex soil-derived bacterial consortia, as a model system for understanding phototroph-heterotroph community assembly.^4^ We cultured the alga with these complex communities and varied the frequency with which exogenous nutrients were supplied. We are able to disentangle the contributions of abiotic factors (dilution frequency or growth period) and biotic factors (the presence of algae) to community assembly. By comparing bacterial community assembly with and without the alga, we show that when dilution frequency is high (i.e., external nutrients are supplied frequently), the presence of algae does not impact bacterial community assembly. However, when dilution frequency is low (i.e., when external nutrients are not supplied frequently), the presence of algae strongly influences community assembly. In a regime where external nutrients are supplied infrequently (low dilution frequencies), algae drive initially distinct bacterial consortia to converge to a more similar taxonomic composition. We show that this convergence is driven by algal net inhibition of specific bacterial taxa in bacterial communities. Direct measurements of interactions are qualitatively consistent with these results.

### The need for model phototroph-heterotroph communities

Given the importance of phototrophic microbes in primary production and sustaining bacterial communities, it has been proposed that phototrophs might recruit specific bacterial taxa to their local environment to enable productive symbioses.^60^ This thinking has led to the idea that phototrophic microbes might locally control bacterial community assembly. However, studies attempting to address this question have yielded conflicting results. For example, incubations of lake water with three phototrophs showed that the assembled bacterial communities at the class level were tightly correlated with the identity of the autotroph.^6^ Other studies on algae and diatoms have found similar results.^30^^,^^38^^,^^42^ Conversely, studies have shown that the identity of the phototroph is only weakly associated with the taxonomy of the associated bacterial consortium.^70^ Further, other works showed that bacterial communities in the phycosphere are weakly taxonomically constrained by the identity of the phototroph to contain certain classes of bacteria such as *Roseobacteria*.^33^^,^^57^ From these studies, it remains unclear when and how photosynthetic microbes impact local bacterial community assembly. Here, we addressed this problem empirically by using naturally derived bacterial communities and the alga *C. reinhardtii*. Leveraging “hybrid” communities comprised natural consortia with domesticated phototrophs afforded us control over the composition of the communities while retaining some measure of the complexity of wild communities.

## Results

### Algal impact on taxonomic composition depends on dilution frequency

In conditions where externally supplied nutrients are limiting, we expect the compounds secreted by the algae to have a significant effect on the bacteria that can cohabit with the algae. Therefore, in a system consisting of algae and a diverse pool of bacteria, if nutrients are supplied externally at a high enough rate, we expect the algae to have little effect on selecting the bacteria and the converse.

**Figure 1:**
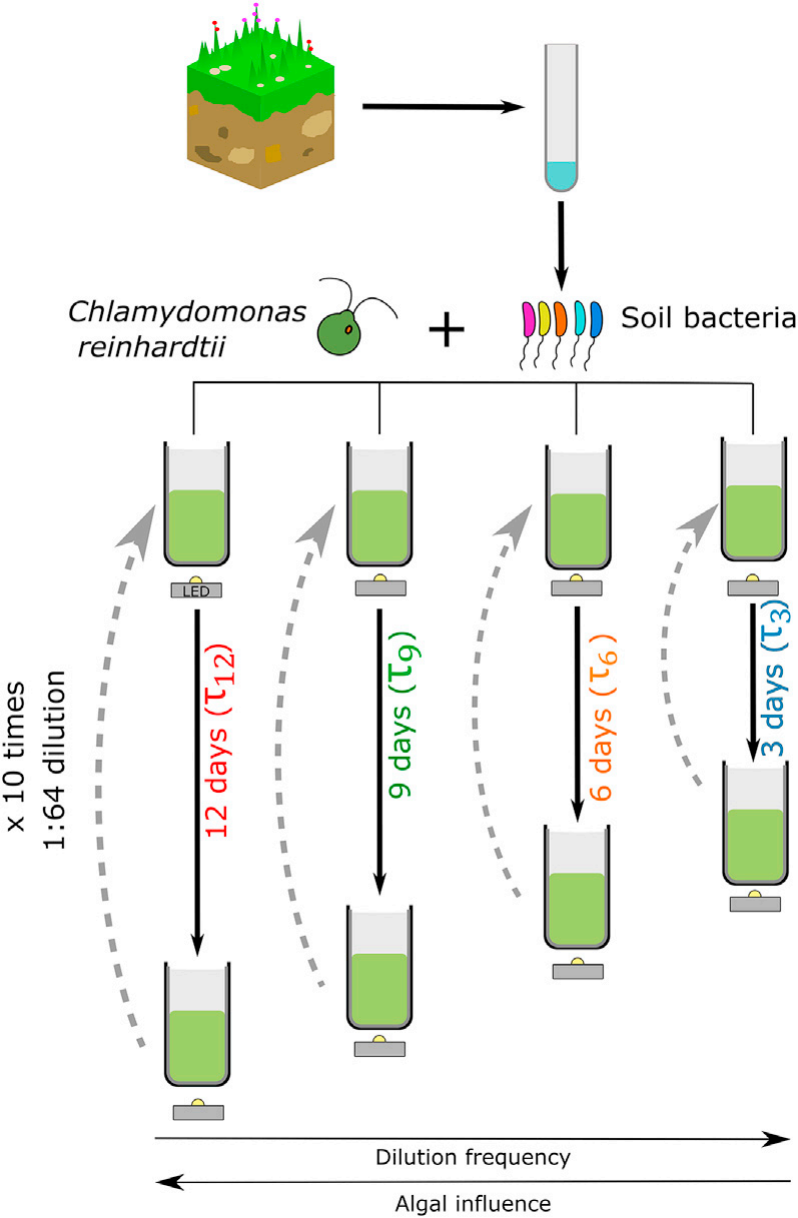
Variable frequency of dilution Soil samples collected from a restored prairie are treated with drugs to remove eukaryotes, including fungi (STAR Methods). The soil bacteria are then mixed with a lab strain of *C. reinhardtii*, UTEX2244, mt+. These co-cultures are illuminated with light/dark (12 h/12 h) cycles and grown for different growth periods—3 days, 6 days, 9 days, and 12 days as indicated. The different growth periods or dilution frequencies alter the frequency of external nutrient supply. The culture volume is 20 mL; they are grown at a temperature of 30°C, with constant stirring in 40 mL capacity glass vials closed with foam stoppers. The approximate generation time of algae is 8 h, allowing the algae to reach saturation in 3 days. At the end of the growth period, the culture is diluted 64-fold into fresh media. Ten such serial dilutions are performed. Therefore, the total culture times are 30 days, 60 days, 90 days, and 120 days for the communities with growth periods of 3 days, 6 days, 9 days, and 12 days, respectively. Each growth period has 5 replicate cultures. Each growth period also has control cultures, where *C. reinhardtii* is not added (not shown in the figure). See also Table S1.

To test this idea, we used soil samples to initiate communities with a diverse pool of bacteria. To maintain uniformity in algae across the different soils, we used a lab strain of the soil-dwelling alga *C. reinhardtii*^58^ as the phototroph. We limited the amount of organic carbon in our media (STAR Methods) to enforce interactions between *C. reinhardtii* and the bacterial pool, and added excess amounts of nitrogen (ammonia) and phosphorous to ensure no limitation occurred for these nutrients. To test our hypothesis, we performed serial dilutions on the communities after allowing them to grow for different growth periods—3 days ($\tau_{3}$), 6 days ($\tau_{6}$), 9 days ($\tau_{9}$), and 12 days ($\tau_{12}$) (Figure 1). In these conditions, the algae reached a saturating density of about 10^7^ cells/mL over a period of about 3 days of growth (inferred from Figure S15). As a result, in the longer growth period conditions, the algae reached saturation after about 3 days and remained at high density for the rest of the growth cycle. In the shorter growth conditions, the algae persisted through the serial dilutions, since they grew to high densities in 3 days (Figures S1 and S18). Since algae continue to excrete nutrients even after reaching saturation,^3^^,^^5^^,^^10^^,^^18^^,^^48^^,^^64^ we expect the impact of *C. reinhardtii* to be stronger in communities undergoing dilution every 12 days and weaker in communities undergoing dilution every 3 days. After they were grown for their respective growth periods, the communities were diluted into media with fresh nutrient supply. We performed 10 rounds of serial dilution for each growth period. We used two soil samples (designated “A” and “B”, STAR Methods) to start the experiments. For each growth period, we had five replicates of communities of each soil sample with *C. reinhardtii* (hybrid communities) and two (for soil sample B) or three (for soil sample A) communities without *C. reinhardtii*, as controls (control communities). A schematic of the experimental design is presented in Figure 1.

**Figure 2:**
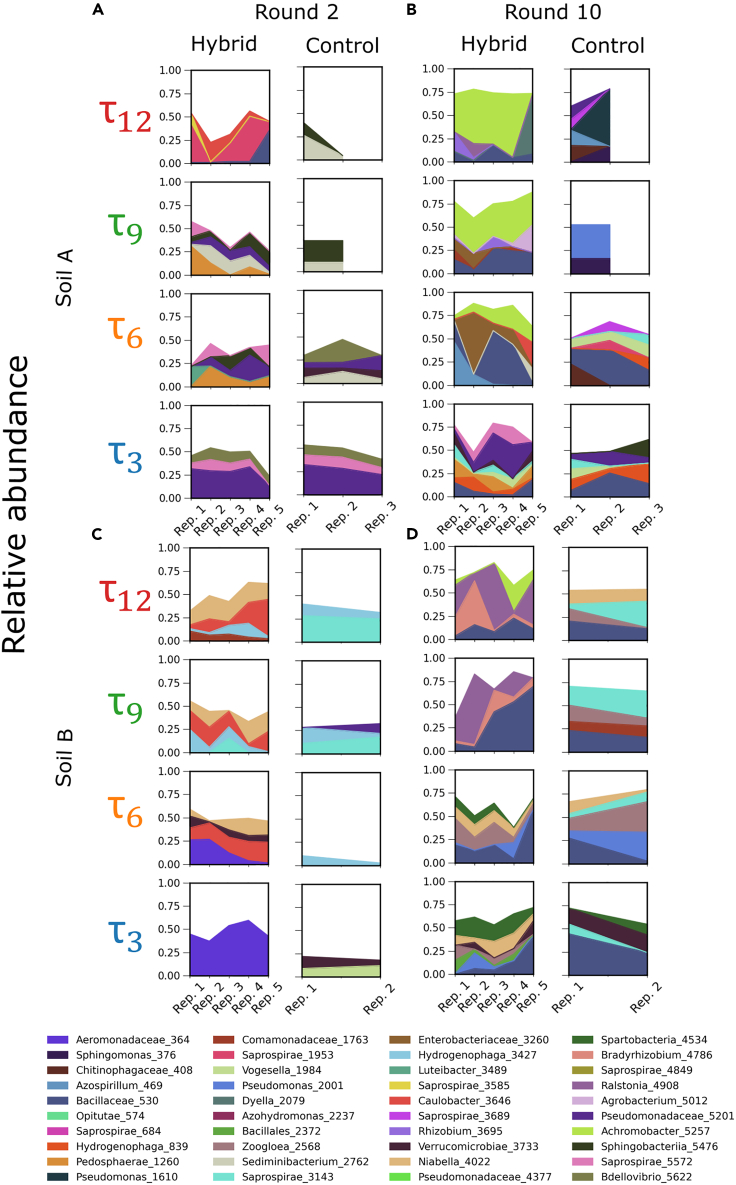
Relative abundance of dominant OTUs at rounds 2 and 10 of serial dilution for replicate hybrid and control communities at all growth periods Dominant OTUs are defined, for illustration purposes only, as those with a relative abundance of at least 20% at any dilution round, soil sample, or growth period. (A and B) Panels A and B show the relative abundance of dominant OTUs in cultures inoculated from soil sample A at rounds 2 and 10, respectively. Rows correspond to growth periods as shown by labels to the left. (C and D) Panels C and D show dominant OTU relative abundance for cultures inoculated from soil sample B at rounds 2 and 10, respectively. In each panel, the first column shows data for hybrid communities, and the second column shows data for the control communities, as indicated by the labels on the top of each column. Ticks on the abscissa demarcate each replicate community in a given treatment; the hybrid communities have 5 replicates and the control communities have either three (soil A) or two (soil B). OTU taxonomic classifications are shown in legend at the bottom. See STAR Methods for more details. See also Figures S2–S4 and S18 and Data S1, S2, S3, and S4.

In order to assess the effect of the algae on the assembly of the heterotrophic communities, we performed amplicon sequencing of the V4 region of the 16S small subunit rRNA gene on the communities after every other round of serial dilution (STAR Methods, Data S1, S2, S3, and S4). From these data, we identified operational taxonomic units (OTUs) in each community using the DADA2 pipeline^13^ (STAR Methods). In Figures 2A and 2B we show the dominant taxa, defined for illustration purposes only as those present in at least 20% relative abundance in any time point or community, for the replicate hybrid and control communities, respectively.

We observe from Figure 2 that in the early dilution rounds, the communities inoculated with different soils were distinct from each other. By dilution round 10, we observed that the hybrid communities inoculated from the same soil had some common dominant OTUs across the 12 days, 9 days, and 6 days growth periods within a soil sample (e.g., Ralsontia and Bradyrhizobium in soil B and Achromobacter in soil A). However, we did not observe this effect in any $\tau_{3}$ control communities or the hybrid communities. This suggests that *C. reinhardtii* had a stronger impact on the selection of the OTUs for the longer growth periods, where external nutrient supply was infrequent. The composition of the communities was also distinct from the composition of the soil used to inoculate the communities (Figure S2). The finding that the dominant taxa were similar in hybrid communities with 6, 9, and 12 days growth periods holds if we defined taxa at the amplicon sequence variant (ASV) level or the genus level (Figures S3 and S4).

From Figure 2, we also observe that the control communities were taxonomically distinct from the hybrid communities, especially at later dilution rounds. For example, the composition of the $\tau_{3}$ hybrid and control communities at round 2 for soil A was very similar. This similarity decreased at round 10 (Figure 2). We quantified this similarity using the Jensen-Shannon divergence (JSD) metric (Figure S5) and found that indeed, the control communities were significantly different from the hybrid communities, and this difference increased with dilution rounds for the longer growth periods (9 and 12 days, Figure S5). This again implies that the presence of *C. reinhardtii* drove the hybrid communities with longer growth periods to be distinct from the control communities. Because the distinction between hybrid and control communities was stronger for the longer growth periods, we infer that the algal impact was stronger in the communities with the longer growth periods, where the frequency of external nutrients supplied was infrequent.

**Figure 3:**
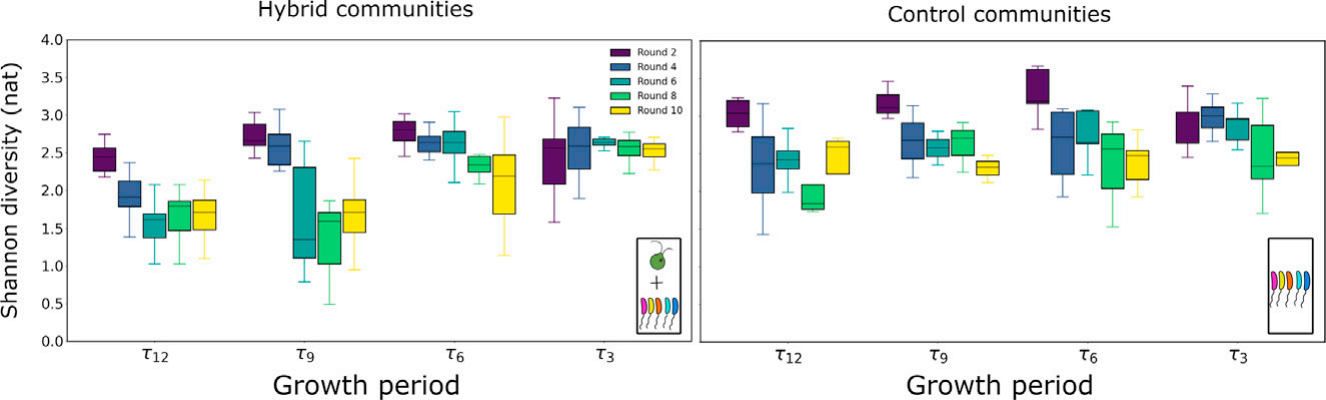
Impact of the growth period and presence of algae on community diversity The distributions of Shannon diversity metrics of the communities across replicates are plotted here as a function of the growth period for the different dilution rounds (black to yellow). The boxes extend from the first to the third quartile, the horizontal line shows the median and the whiskers represent 1.5 times the inter-quartile range (first–third quartile). For the $\tau_{9}$ and $\tau_{12}$ hybrid communities, the diversity drops significantly by round 10 compared to their respective control communities at round 10 (p value 0.05 and 0.03, respectively, for the null hypothesis that the diversities of the control communities is the same as the hybrid communities). Similarly, diversity in the $\tau_{3}$ hybrid communities is higher than $\tau_{12}$ hybrid communities (p value of 0.0002 with the null hypothesis that the diversity of the hybrid $\tau_{3}$ communities is the same as the hybrid $\tau_{12}$ communities). For shorter growth periods, there is no difference in diversity between control and hybrid communities (p value of 0.9 for the null hypothesis that $\tau_{3}$ control communities have the same diversity as the $\tau_{3}$ hybrid communities.) The Kolmogorov-Smirnov test was used to compute the p values. See also Figure S1.

To globally assess the effect of *C. reinhardtii* on bacterial community assembly, we computed the Shannon diversity index for all communities in our experiment. We found that for hybrid communities with longer growth periods, the diversity decreased with rounds of dilution (Figure 3, left panel). Similarly, $\tau_{3}$ hybrid communities had higher diversities than $\tau_{12}$ and $\tau_{9}$ hybrid communities by dilution round 10. However, the diversity of the $\tau_{3}$ hybrid and control communities was not significantly different by round 10 (Figure 3, compare $\tau_{3}$ left and right panels). This indicates that it was the presence of *C. reinhardtii*, whose impact was more strongly felt in the hybrid communities with longer growth periods, that was responsible for the decline in diversity of the hybrid communities with longer growth periods.

### Dimensionality reduction shows algae drive convergence of bacterial community composition

To see if the impact of algae (biotic factor) and dilution frequency (abiotic factor) on community assembly could be distinguished, we performed principal component analysis (PCA) on the relative abundance data. We included all hybrid and control communities, at all growth periods and dilution rounds in this analysis. Data from the initial soil inoculates were also included. The reads mapping to rare taxa (STAR Methods), chloroplast, and mitochondria were removed. Using a permutation approach, we found that only the largest two eigenvalues exceeded the noise in our dataset (STAR Methods). Therefore, we studied the dynamics of our communities along the first two principal components, which accounted for 14.6% and 10.4% of the variance of the data, respectively.

**Figure 4:**
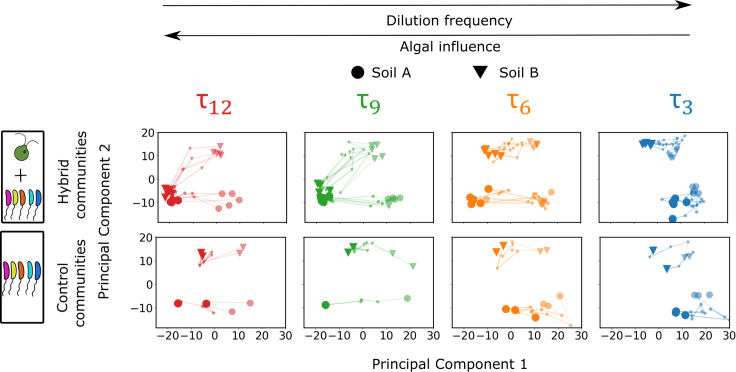
Principal components analysis reveals that the presence of algae mediates community convergence for longer growth periods Principal components analysis (PCA) was performed on the abundance data for all hybrid and control communities. Rare taxa, defined as those below a threshold relative abundance in all communities and time points, were removed. This method sets a cutoff of 0.027% for relative abundance. Removing rare taxa simplifies downstream analysis and does not affect our results (Figure S6). A small number is added to the rest of the dataset to remove zeroes and the relative abundances for each community are center-log transformed. PCA was performed on this matrix and two components were deemed significant using a permutation approach (STAR Methods). The two components explain 14.6% and 10.4% of the variance in the data, respectively. A single PCA was performed for all communities but for visualization, we show growth periods, hybrid, and control communities separately. The top row of panels shows the hybrid communities (those with *C. reinhardtii* as indicated at the left) with growth period decreasing from left to right and the bottom row of panels show the control communities (those without *C. reinhardtii*). In each panel, circles indicate soil A and triangles indicate soil B. The mid-size markers indicate the communities at Round 2 and the largest markers indicate the communities at Round 10. The smallest markers indicate the communities at Rounds 4, 6, and 8. Colors denote growth periods as indicated above each column. Black arrows along the top indicate the frequency of external nutrient supply increasing from left to right and increasing algal influence on community assembly from right to left. See also Figures S6–S10.

We present projections of community composition onto the first two principal components in Figure 4. Although a single PCA analysis was performed on all communities at once, for the purpose of visualization we split up the data by the presence/absence of *C. reinhardtii* and growth period.

Two main features are clear from Figure 4. First, it is apparent that at round 2, the two soil samples were taxonomically distinct, both in the hybrid and control communities (compare the intermediate-sized translucent circles and triangles in all panels of Figure 4). However, with dilution rounds, the $\tau_{9}$ and $\tau_{12}$ hybrid communities moved closer to each other, with communities from soil A moving along the first principal component (PC1), and communities from soil B moving along PC1 and principal component 2 (PC2). We refer to the fact that the distance between communities from soil samples A and B rapidly declines with dilution rounds in the $\tau_{12}$ and $\tau_{9}$ hybrid conditions as “convergence”. Second, the control communities (Figure 4 bottom panel) did not converge, but they did move along PC1 as did all hybrid communities irrespective of the presence of algae. These features of the community dynamics were also observed when we performed other dimension reduction methods on the data (Figures S9 and S10) or used the data without removing rare taxa (Figure S6 C). Next, we sought to dissect how the abundance dynamics within the bacterial consortia drove these two outcomes.

### Convergence along principal component 2 arises from net inhibition of bacteria in the presence of algae

**Figure 5:**
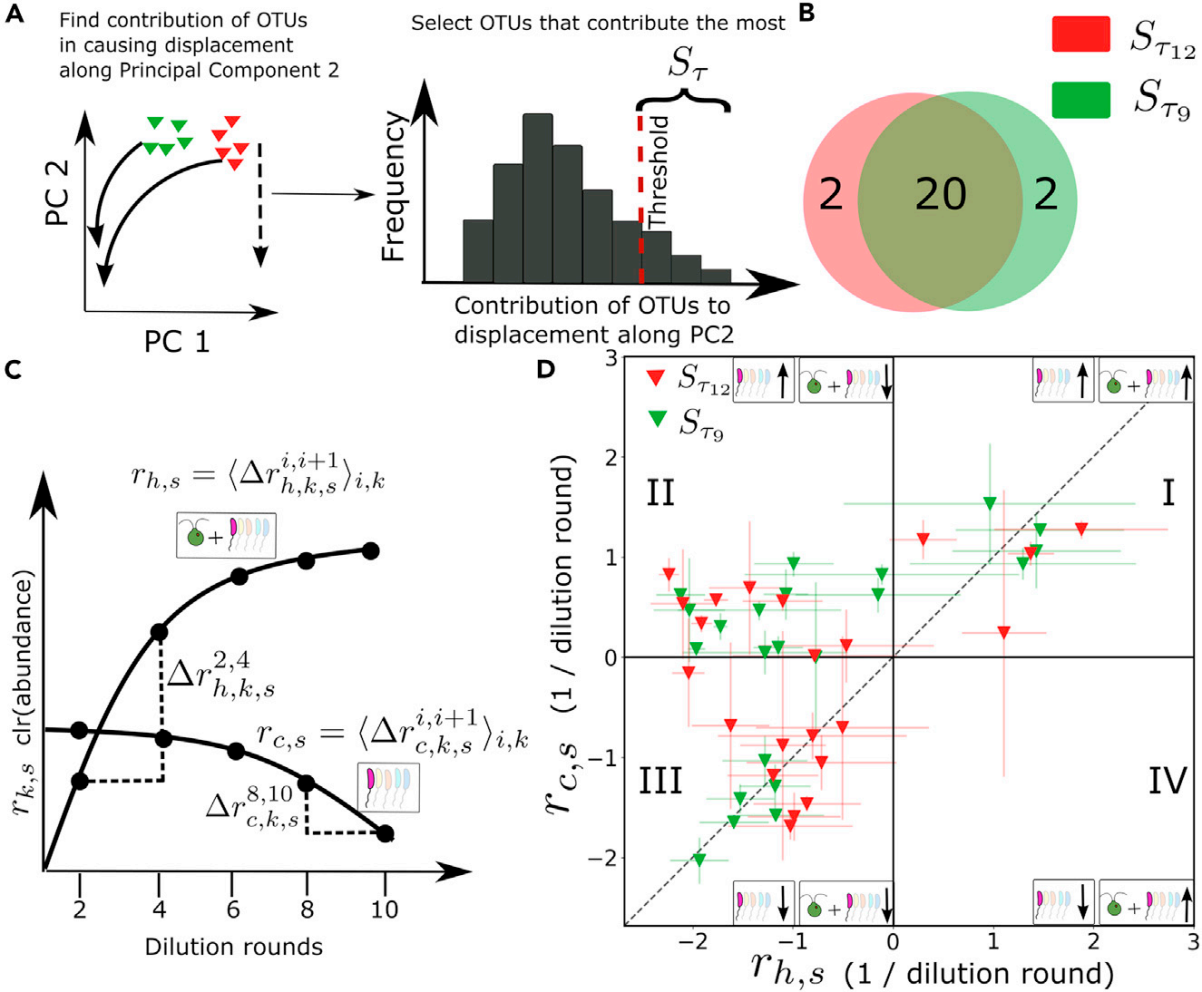
Hybrid community convergence arises from inhibition of bacterial taxa in the presence of algae (A) The contribution of each OTU to the displacement along PC2 is found for $\tau_{12}$ and $\tau_{9}$ hybrid communities from soil B (triangles). A histogram of the contribution of each taxon to displacement along PC2 is computed and the taxa with contributions above a threshold are selected ($S_{\tau }$). (B) A Venn diagram comparing the identity of taxa selected by the procedure in panel A for both 9 and 12 days growth periods. (C) To compare the dynamics over the rounds of serial dilution, enrichment of these algae-driven taxa is computed in both hybrid and control communities. We denote the center log-transformed relative abundance of OTU *s*, in replicate *k*, at time point *i* for a hybrid community as $r_{h,k,s}^{i}$. The change in relative abundance between two adjacent time points is then $\Delta r_{h,k,s}^{i+1,i}=r_{h,k,s}^{i+1}-r_{h,k,s}^{i}$. For each OTU we then average these changes across time points and replicates: $r_{h,s}={\left\langle \Delta r_{h,k,s}^{i+1,i}\right\rangle }_{k,i}$. For each OTU we perform the same analysis in control communities to estimate the enrichment in the absence of algae ($r_{c,s}$) (STAR Methods). (D) The enrichment rates in the hybrid community ($r_{h,s}$) are plotted against the rates in control communities ($r_{c,s}$) for each OTU selected in panel A. The error bars indicate standard deviation over replicates. Colors indicate the growth period used to compute the enrichment. See the main text for an interpretation of each quadrant labeled with Roman numerals. See also Figures S6 and S11–S13.

Convergence occurred when the $\tau_{12}$ and $\tau_{9}$ hybrid communities of soil sample B moved down along PC2 over the rounds of serial dilution to become taxonomically similar to soil sample A by dilution round 10 (Figure 4, $\tau_{12}$ and $\tau_{9}$ top row panels). Since this convergence only occurred for the hybrid communities with long growth periods, the convergence was caused by *C. reinhardtii*. We wanted to understand how the algae affected the heterotrophs over the dilution rounds to cause this convergence. To find the heterotrophs causing motion along PC2, we used the eigenvector loadings along PC2 to find the contribution of each OTU to the motion along PC2 for the $\tau_{12}$ and $\tau_{9}$ hybrid communities of soil sample B. From the distribution of contributions, we selected those OTUs that had the highest contribution (Figure 5A, STAR Methods) to the displacements. Since we have established that biotic factors, i.e., *C. reinhardtii* causes the displacement along PC2, we call these bacterial taxa, through which the effect of the algae is manifested, the “algae-driven taxa”. We found that these sets of taxa for hybrid communities with growth periods $\tau_{9}$ ($S_{\tau_{9}}$) and $\tau_{12}$ ($S_{\tau_{12}}$) had more than 90% taxa in common; i.e., *C. reinhardtii* affected the same taxa for both growth periods to displace the communities along PC2 (Figure 5B). In other words, changes in the relative abundances of the same OTUs caused motion along PC2 for both the $\tau_{9}$ and $\tau_{12}$ hybrid communities in soil B.

This simple analysis told us which taxa are driving the convergence along PC2 but did not reveal the dynamics of these taxa over the rounds of serial dilution. Therefore, we sought to understand how these taxa ($S_{\tau_{12}}$ and $S_{\tau_{9}}$), that drove convergence in hybrid communities were changing in time over the course of the enrichment experiment. To obtain their dynamics over the dilution rounds, we found the differences in the center log transformed relative abundances (*r*) of the algae-driven taxa over the successive dilution rounds in both hybrid and control communities. For each algae-driven taxa, i.e. each taxon *s* in $S_{\tau_{12}}$ and $S_{\tau_{9}}$, we computed the average net relative enrichment of the center log transformed relative abundance, *r*, over the dilution rounds, in the presence of algae, i.e. in hybrid communities, $r_{h,s}$, where *h* denotes hybrid communities, and in the absence of algae, i.e, in the control communities, $r_{c,s}$, where *c* denotes the control communities (STAR Methods). The sign of $r_{h,s}$ or $r_{c,s}$ tells us, on average, whether the relative abundance of OTU *s* is increasing or decreasing over dilution rounds in the presence and absence of algae respectively. As sketched in Figures 5C and 5A given taxa could have different relative abundance dynamics in the presence and absence of algae.

By plotting $r_{h,s}$ versus $r_{c,s}$ we can gain insight into the impact of algae on the relative abundance dynamics of each algae-driven taxon *s*, Figure 5D, where each point corresponds to a single taxon from either $S_{\tau_{9}}$ (green) or $S_{\tau_{12}}$ (red). Note that enrichment here is computed on relative abundance, so we cannot say if a given taxon is going up or down in absolute terms, an important limitation of this analysis. The diagonal dashed line represents the case where the enrichment of the taxa is the same in both the hybrid and control communities. Algae have no effect on the relative enrichment of the taxa on this line because the enrichment is the same irrespective of the presence of algae. In the first quadrant (I), the relative enrichment is positive for both control and hybrid communities, implying that the taxa in this quadrant increase in relative abundance over dilution rounds in both the control and hybrid communities. In the second quadrant (II), the relative enrichment is positive for control communities, and negative for hybrid communities. Taxa in this quadrant increase in relative abundance in the absence of the algae but decrease in relative abundance in the presence of algae, indicating that they are preferentially inhibited in the presence of *C. reinhardtii*. Note that if a taxon decreases in relative abundance in the presence of algae it need not be directly inhibited by the algae, it could be that other strains increase in absolute abundance driving down the relative abundance of the target taxon. In the third quadrant (III), the relative enrichment is negative for both the hybrid and control communities. The taxa in this quadrant decrease in relative abundance both in the presence and absence of algae. However, the abundance of taxa above the dashed line decreases more strongly in the presence of the algae, because above the dashed line, the relative enrichment is more negative in the hybrid communities than in control communities. In the fourth quadrant (IV), the relative enrichment is positive for the hybrid communities and negative for control communities, implying that the taxa present here increase in relative abundance in the presence of algae. This could occur either by the algae driving up the absolute abundance of that taxon or suppressing other taxa in the community.

We observe that most of the algae-driven taxa ($S_{\tau_{12}}$ and $S_{\tau_{9}}$) were in quadrants II and III, where the algal impact is net suppression of relative abundances. The taxa that contributed the most to the motion along PC2 dominantly occupy the second quadrant of the enrichment plot, for both the $\tau_{12}$ and $\tau_{9}$ hybrid communities, where the presence of algae strongly suppressed their enrichment. In quadrant III, the taxa located above the diagonal are more strongly suppressed in the hybrid communities compared to the control communities. The almost complete absence of taxa in quadrant IV, where enrichment is positive in the hybrid communities but negative in control communities, indicates that taxonomic convergence did not occur due to the same taxa increasing in relative abundance. The dominant effect of the presence of algae was therefore to suppress these algae-driven taxa. In other words, the presence of *C. reinhardtii*, directly or indirectly, caused these algae-driven taxa to decrease in relative abundance over the dilution rounds. We emphasize here that these results are only for the algae-driven taxa that drive motion along PC2. This suppression resulted in the motion of soil B communities along PC2, driving convergence between soil A and B communities at growth periods of 9 and 12 days. Effectively, hybrid communities of soil sample B became more similar to soil sample A by reducing the relative abundances of some OTUs. It is important to note that because we are only looking at the relative abundances of the taxa, it is possible that the observed decrease in the relative abundances could be due to the enrichment of other taxa. Further, we cannot interpret Figure 5D as characterizing direct interactions between algae and any member of the community. Instead, this analysis characterized the global impact of the presence of algae on the relative abundances of each OTU of interest, but these changes may be mediated by indirect effects or emerge from networks of interactions in the community. We address this problem in the following using experiments with isolates.

The finding that algae inhibited specific OTUs in soil B to drive convergence with soil sample A makes sense in light of the results in Figures 2, 3, 4, and S1. First, a close examination of the dominant OTUs in $\tau_{12}$ and $\tau_{9}$ hybrid communities at round 10 in soil samples A and B revealed that the dominant taxa are not the same between these two communities (Figure 2B, hybrid column). As a result, convergence in Figure 4 $\tau_{12}$ and $\tau_{9}$ hybrid communities arose not from algae recruiting similar dominant taxa, but from the net inhibition of taxa that were unique to soil B. In addition, the finding that algae preferentially inhibit bacteria in hybrid communities (Figure 5D) is consistent with the loss of diversity in the hybrid communities (Figure 3) at longer growth periods. Further, measurements of optical density (OD) and chlorophyll fluorescence (Figure S1) revealed that the $\tau_{3}$ hybrid communities have a higher OD per chlorophyll content compared to the $\tau_{12}$ hybrid communities, indicating that there were more heterotrophs per algae in the $\tau_{3}$ hybrid communities compared to the $\tau_{12}$ hybrid communities, consistent with the observed loss of diversity in Figure 3.

In contrast to the long growth period communities, hybrid communities with a 3 days growth period ($\tau_{3}$) did not converge (Figure 4). We concluded that the longer growth periods drive this convergence, but it is reasonable to ask whether the 3 days hybrid communities simply did not have enough time to converge due to shorter total incubation time: 10 rounds × 3 days–30 days, versus 120 days in the 12 days growth period experiment. To interrogate this possibility further we first examined the dynamics of the $\tau_{3}$ hybrid communities from soils A and B. Unlike communities at longer growth periods, we found that the distance between $\tau_{3}$ hybrid communities from the two soil samples along PC2 increased over dilution rounds, whereas for the 9 days and 12 days growth periods, this distance decreased over dilution rounds (Figure S11). Second, we analyzed the dynamics of $S_{\tau_{12}}$ and $S_{\tau_{9}}$ taxa in the $\tau_{3}$ communities using the relative enrichment approach developed above. In contrast to our results in Figure 5D, where the taxa were predominantly on the left half of the plot, where they were suppressed in the presence of *C. reinhardtii*, in the $\tau_{3}$ and $\tau_{6}$ communities we found that the algae-driven taxa were predominantly along the diagonal of the enrichment plot (Figure S12) indicating that *C. reinhardtii* did not impact the bacterial enrichment rates significantly in these conditions. This suggests that the soil A and soil B $\tau_{3}$ hybrid communities would not converge even given additional rounds of serial dilution. We propose that the reason $\tau_{3}$ hybrid communities do not converge is that over just 3 days *C. reinhardtii* has significantly less time to impact bacterial growth. Specifically, we estimated the doubling time of the algae in these conditions to be approximately 8 h. At this growth rate, it took the algae approximately 3 days to reach saturation after each round of 64-fold dilution. In contrast, during a 12 days growth period, algae reached saturation after approximately 3 days and remained at high levels for the remaining 9 days. We expect that during this 9-day period, algae continued to excrete nutrients and other compounds^3^^,^^5^^,^^10^^,^^18^^,^^48^^,^^64^ potentially impacting bacterial community assembly. Collectively, these results support the idea that the 3 days hybrid communities are unlikely to converge even with additional rounds of serial dilution.

### Dynamics along PC1 are driven by abiotic factors

From Figure 4 we observe that all communities, irrespective of the presence of the algae, moved along PC1. Further, the actual displacement along PC1 depended on the dilution frequency, i.e., the frequency of supply of external nutrients. The $\tau_{3}$ communities, both hybrid and control, were displaced much less along PC1 compared to the $\tau_{12}$ communities, both hybrid and control (Figure S13). Because the motion along PC1 was independent of the presence of algae but depended on the growth period, we hypothesize that the motion along PC1 was caused by abiotic factors, in particular, the dilution frequency.

To confirm that the presence of algae did not impact the dynamics along PC1, we performed the following test. Using the eigenvector loadings along PC1, we found the contribution of each OTU to the displacement along PC1 for each community. From these, we selected the major contributors using the cumulative distribution of the contributions (as in Figure 5A, STAR Methods). Using this approach, we identified the taxa-driving dynamics along PC1 for each community. We performed a statistical test with the null hypothesis that the taxa contributing to motion along PC1 for a given soil sample and growth period were equally likely to be selected in the control and hybrid communities. We found that we were not able to refute the null hypothesis (STAR Methods).

We conclude that the same taxa cause motion along PC1 in both the hybrid and control communities, indicating that, statistically, there is no evidence that algae drive motion along PC1. This means that motion along PC1 was caused by an abiotic factor, the frequency of external nutrient supply, or growth period, common to the hybrid and control communities. We denote the taxa that drive motion along PC1 in both hybrid and control communities, as “dilution-frequency driven” taxa.

### Algal impact on bacterial isolates varies

Our analysis of relative abundance dynamics in the enrichment experiment suggested that the dominant effect of algae was to suppress bacterial taxa responsible for motion along PC2 in the long growth period conditions (Figure 5D). As discussed previously, this measurement did not directly characterize interactions between algae and the associated bacterial taxa. To directly elucidate the types of interactions driving dynamics in our communities, we isolated 21 unique bacterial strains from the communities (See STAR Methods and Data S8). These strains represented the taxonomic diversity of the assembled communities well, spanning all four quadrants of the enrichment plot (Figure S14). Further, two of these isolates (Bacillacea 530 and Ralstonia 4908) were dominant taxa as captured by our amplicon sequencing measurements (Figure 2).

**Figure 6:**
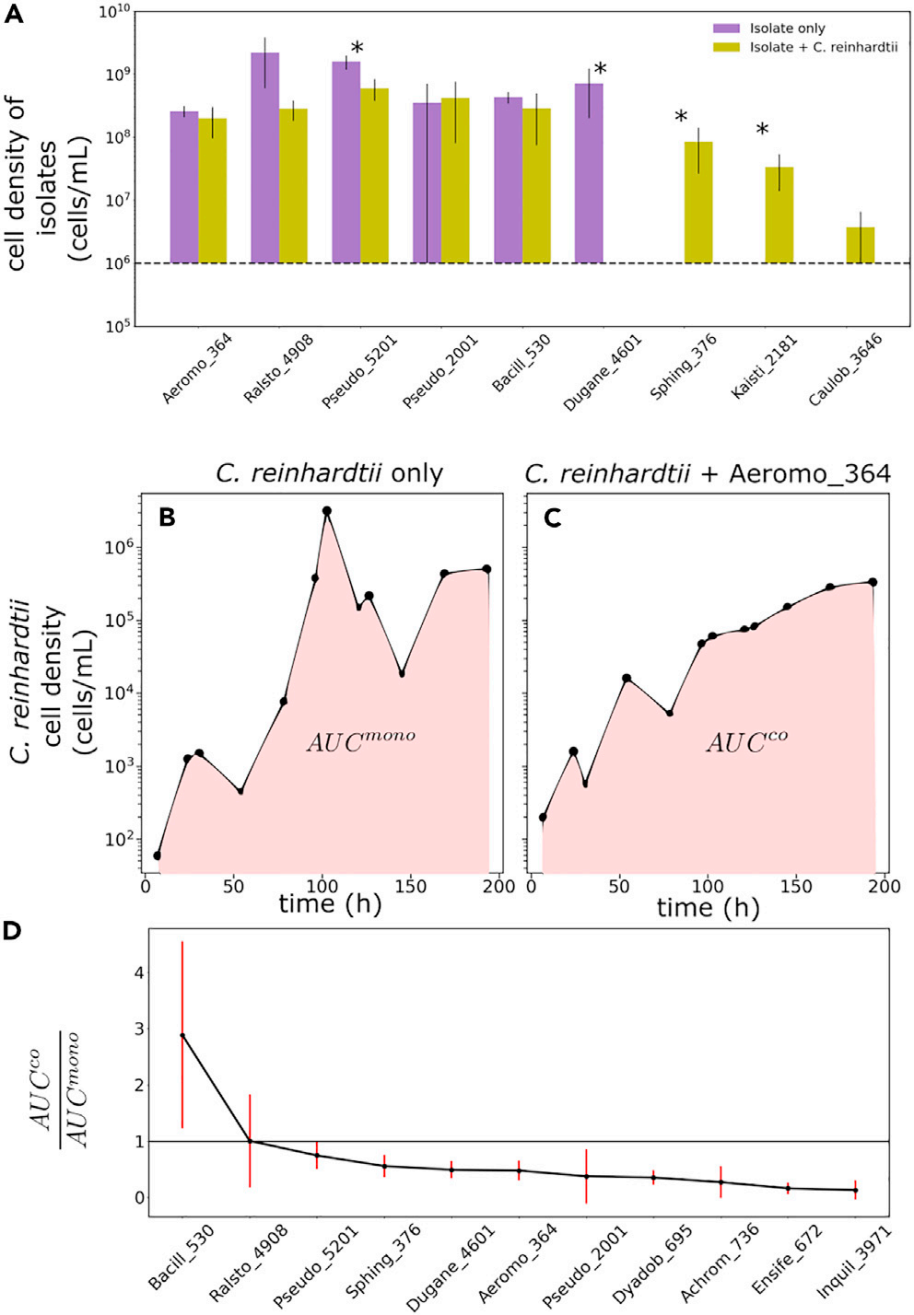
Bacteria inhibit algal growth but can be either positively, negatively, or neutrally affected by algae (A) Panel A shows the impact of algae on bacterial growth. (B–D) Panels B–D show the impact of bacteria on algal growth. A Characterizes the impact of algae on the growth of bacteria. Each bacterial isolate was grown in co-culture with *C. reinhardtii* in 2–5 replicates. The error bars correspond to standard deviations across replicates. After 14 days bacterial density was assayed by plating (STAR Methods). Bars indicate measured bacterial density with (yellow) and without (purple) algae. The first six isolates were either inhibited by algae or algae had no impact on growth. The last three bacterial isolates, which grow to a density below the detection limit of $1\times 10^{6}$ cells/mL (represented by the horizontal dashed line) on glucose, grow better in the presence of algae than in the absence. The stars indicate the cases where growth with and without the algae are significantly different as tested by the Kolmogorov Smirnoff test. B shows an example trace of cell density of *C. reinhardtii* grown alone. Densities are measured by flow cytometry. The shaded area represents the area under the curve (AUC) for the monoculture, $AUC^{mono}$. C shows an example trace of cell density of *C. reinhardtii* grown in co-culture with a bacterial isolate (Aeromo 364). The shaded region represents the AUC for co-culture, $AUC^{co}$. D shows the ratio $\frac{AUC^{co}}{AUC^{mono}}$ for different bacterial isolates from our communities. This ratio is larger than 1 for bacteria that enhance algal growth relative to monoculture and < 1 for bacteria that inhibit algal growth. Each co-culture and the monoculture were run in triplicate, and errors in $\frac{AUC^{co}}{AUC^{mono}}$ were computed via error propagation from the standard deviation across the three replicates. With the exception of Bacill 530, all other isolates have either an inhibitory or neutral effect on the growth of the algae. See also Figures S14 and S15 and Data S8 and S9.

Since we observed primarily inhibition of the algae-driven taxa in our enrichment analysis of the sequencing data (Figure 5D), we first wanted to directly assay the effect of algae on bacterial growth. To accomplish this, we grew each isolate on its own and in the presence of algae in conditions identical to the enrichment experiment (STAR Methods). We assayed the final bacterial carrying capacity after two weeks of co-culture, quantifying bacterial abundances via measurements of colony-forming units (CFUs). To assess the impact of algae on bacteria we compared the carrying capacity of some of our bacterial isolates with and without the algae. We observe that for 5 of the 9 isolated strains, there was no significant effect of the algae (Figure 6A). In two cases, the presence of the algae significantly inhibited the growth of the bacterial isolates. Algae significantly enhanced the growth of two other isolates.

We wanted to check whether our measurements of algal impacts on bacterial growth were consistent with our analysis of the sequencing data. For example, we might naively expect that the taxa present in quadrant II of Figure 5D to be inhibited by the algae. Therefore, we performed an identical analysis to Figure 5D for OTUs that correspond to our library of isolates (Figure S14).

Of the 9 bacterial isolates used here, Aeromo 364, Ralsto 4908, Pseudo 5201, Kaisti 2181, and Caulob 3646 belong to the set of algae-driven taxa ($S_{\tau_{9}}$ and $S_{\tau_{12}}$). Aeromo 364 and Ralsto 4908 were close to the diagonal on the enrichment plot (Figure S14), which was consistent with the observation here that the abundances of these two taxa were not significantly impacted by the presence of algae (Figure 6A). Similarly, Pseudo 5201 is located in the third quadrant of the enrichment plot in Figure S14, above the diagonal, implying that it was inhibited more strongly in the presence of algae than in its absence, consistent with the observation that it was inhibited by the algae. Also, the enhanced growth of Kaisti 2181 in the presence of algae was observed in our enrichment analysis and our interaction measurement. In contrast, algae inhibited Caulob 3646 in the enrichment analysis (quadrant II in Figure S14) but direct measurements of interactions revealed no effect of algae on its growth.

In contrast, Pseudo 2001, Bacill 530, and Dugane 4601 are part of the dilution-frequency driven taxa, i.e., they caused motion along PC1 for all communities irrespective of the presence of the algae. One would therefore expect that the presence of algae has no effect on their enrichment dynamics. This is indeed observed when direct interactions were measured for Pseudo 2001 and Bacill 530 (Figure 6A). However, Dugane 4601 did not follow this trend, being strongly suppressed in the presence of algae. Similarly, Sphing 376, which is neither an algae-driven nor a dilution-frequency-driven taxa, was strongly suppressed in the hybrid communities, whereas the direct measurement of the interaction revealed that it grew better in the presence of algae.

On the whole, though they do not reveal the mechanisms, the interaction measurements corroborate the sequencing measurements. However, there are exceptions, for which there is not a clear interpretation of the interactions shown in Figure 6A in terms of the location of each isolate on the enrichment plot and their contribution to motion along PC1. The reason for this discrepancy with the co-culture data (Figure 6A) may be that the pairwise interaction is not representative of the emergent dynamics of this OTU and the community context matters qualitatively, or that our isolate is a strain with different ecological interactions than the dominant OTU assigned to that ASV,^28^ which is a disadvantage of using OTUs over ASVs.

### Bacterial isolates inhibit algal growth

In the previous section, we examined the effect the algae had on the bacteria. Now, using the library of isolates we wanted to understand the effect of bacteria on the growth of *C. reinhardtii*. To study this, we grew *C. reinhardtii* with each isolate individually in conditions identical to those of the enrichment experiment (STAR Methods). We included an algae-only control. Samples were collected from each vial approximately daily and algal abundances were quantified by flow cytometry. Examples of algal abundance dynamics with and without a bacterial isolate are shown in Figures 6B and 6C. To quantify the impact on the algal growth of a bacterium we computed the area under the growth curve (AUC)^31^ for algae alone and in the presence of bacteria. If the AUC is lower for algae in the presence of bacteria (AUC^co^) than it is for algae alone (AUC^mono^) this indicates inhibition of algal growth by bacteria via increased lag phase duration, decreased growth rate, or decreased carrying capacity or a combination of any of these. Therefore, to assess the overall impact of bacteria on algal growth we computed the ratio of (AUC^co^)/(AUC^mono^). If this ratio is >1, it indicates bacterial facilitation of algal growth, if it is <1, it indicates suppression. Figure 6D shows that only one of the isolates we assayed facilitated algal growth and all others acted to inhibit algal growth or were neutral. The isolate Ralsto 4908 had no significant impact on algal growth and we find it was enriched in the presence of algae during the enrichment experiment.

### Bacterial carbon use preferences predict enrichment rates

While our enrichment analysis (Figure 5D) suggests algae predominantly inhibit the algae-driven bacteria in hybrid communities, other taxa do rise in abundance in the long growth period conditions over 10 rounds of dilution (Figure 2). This suggests that some of these taxa might be consuming algal exudates. A direct measurement of bacterial uptake of algal exudates is beyond the scope of this work, so we decided instead to see if the catabolic phenotypes of our isolates were related to their dynamics during enrichment. To investigate this we assayed the growth of each isolate in our library on carbon compounds excreted by *C. reinhardtii*. We determined the compounds excreted by the alga in monoculture using untargeted metabolomics on spent media (STAR Methods). From these data, we selected 6 organic carbon compounds that accumulated in time in algal monoculture and tested the growth of all 21 isolates on them and glucose (Figure S16A). To see if these resource preferences had any relation to the dynamics of taxa in our experiment we asked whether or not these resource preferences were statistically related to the enrichment ($r_{h,s}$ and $r_{c,s}$) for each isolate in the library. To do this we performed a regression analysis to predict the enrichment, $r_{h,s}$ and $r_{c,s}$ using whether or not each isolate grows on each carbon source as a binary independent variable. This analysis (see STAR Methods, Figure S17) showed that for the isolates in the library, $r_{h,s}$ for $\tau_{12}$ and $\tau_{9}$ was predictable ($R^{2}\approx 0.3-0.4$) based on carbon resource preferences, but that these preferences had no predictive power for $r_{h,s}$ measured in the $\tau_{3}$ or $\tau_{6}$ hybrid communities (Figure S16). Similarly, the carbon consumption preferences had no predictive power on the enrichment of the isolated taxa in control communities, $r_{c,s}$ (Figure S16). Carbon sources excreted by the algae had significant coefficients while glucose did not (Figures S17C–E). Although weak, the fact that the catabolic abilities of the bacteria have some predictive power on their dynamics suggests that the dynamics of bacteria in the long growth periods are influenced by the catabolic capabilities of each OTU, and that the consumption of algal exudates becomes more important as the frequency of the supply of exogenous nutrients decreases.

## Discussion

Using laboratory enrichment experiments where we varied the dilution frequency and the presence of a phototrophic alga, we demonstrated the relative contributions of abiotic and biotic factors to community assembly. We showed that phototrophs impact community assembly when dilution frequency or external nutrient supply frequency is low, suggesting that high levels of allochthonous nutrients should serve to decouple phototrophic microbes from their heterotrophic partners. When initially diverse bacterial consortia from distinct soil samples are grown in long growth periods (9 or 12 days) we find that the presence of the algae drives a convergence in community composition. Thus, phototrophs appear to engineer their local microbiota when exogenous nutrient supply frequency, controlled here, by the dilution frequency is diminished. Analysis of changes in relative abundances of bacterial taxa in the presence and absence of the algae shows that this convergence emerges from net inhibition of bacterial abundances in the presence of algae. Direct measurements of the impact of algae on bacteria confirm this coarse picture but also show that some bacteria are dependent on algal exudates for growth. Direct measurements also show that the bacterial isolates have a negative impact on algal growth, which we speculate could be a reason for the algae to inhibit taxa.

One striking result of our study is the convergence of bacterial communities in environments with low dilution frequencies, or low external nutrient supply rates when algae are present. At high dilution frequencies, glucose consumption ability is likely the dominant factor for community assembly, while at low dilution frequencies, the presence of algae is important. Convergence of community composition despite diverse initial compositions supports the idea that the local environment strongly controls assembly.^26^^,^^43^ However, our result extends this insight by demonstrating biotic contributions to the environment, namely the presence of specific metabolic phenotypes such as phototrophy, can themselves alter the environment to determine emergent community composition. Our result points to the idea that assembly might be best understood by considering not just abiotic factors but also the traits of those taxa present in the community.

Our enrichment analysis shows the net algal impact on the algae-driven taxa, which could be direct, or indirect via intermediate taxa. This analysis of enrichment rates for bacterial taxa with and without algae provides a clear picture of the net impact of the presence of algae on the changes in relative abundances of bacteria. While it is widely appreciated that phototrophs can recruit specific bacterial strains via nutrient exchanges^60^ our results indicate that negative interactions are important for phototrophic impacts on bacterial community assembly. Overall, our data suggest that algal-mediated convergence of bacterial community composition is dominated by net negative impacts on bacterial abundances (Figure 5D). However, our experiments with isolates indicate that some taxa in the community are likely relying on algal exudates to sustain growth (Figure 6D).

The precise interplay between the net inhibition of bacteria and the algal recruitment of bacterial taxa via nutrient excretion remains unclear. While we show that for convergence, suppression in the presence of algae is the dominant mechanism, our statistical analysis of bacterial catabolic phenotypes and enrichment rates suggests that the ability of bacterial taxa to consume algal exudates is important for determining their dynamics at low dilution frequency. Further, our inability to isolate dominant taxa could hint at a high level of inter-dependency between these taxa and the algae, which would make isolating them challenging. However, because our isolate library does not include most of the dominant taxa from different dilution rounds we cannot determine whether those taxa that rise in relative abundance in later rounds of the long-growth period treatments are doing so because they can exploit autochthonous resources provided by the autotroph or not. For instance, the drop in pH over 12 days of growth (see STAR Methods) could play an important role in the assembly of the communities. Future work should focus on dissecting the interplay between these processes at a community level by using synthetic communities.^20^ Further, it should be noted that while varying the dilution frequency certainly changes the frequency of external nutrient availability, it also affects other properties of the culture such as time in stationary phase, which could play a role in the community assembly and dynamics.

The mechanisms through which the phototrophs and heterotrophs interact in our experiment are not known. While the regression analysis indicates that consumption of carbon sources secreted by the algae could play a role in selecting taxa, we find that the dominant effect of the algae is to inhibit bacteria to cause community convergence when nutrients are scarce. This suppression could arise via various mechanisms. One possibility is that acidification of the medium, potentially caused by algae consuming ammonia as a nitrogen source^59^ (STAR Methods), might inhibit certain taxa. For instance, pH has been shown to drive the assembly of specific bacteria in soil across spatial scales^21^^,^^39^^,^^56^^,^^66^ and determine diversity.^69^ Other reasons could be competition for resources as evidenced by the mutual inhibitory interactions (Figure 6), parasitism,^55^ or oxidative stress.^52^ While our control communities without algae show that convergence occurs only in the presence of algae, inter-bacterial interactions could play a role in community assembly. Further work needs to be done to decipher these interactions.

Our work provides important context for studies seeking to understand the role of phototrophs^30^^,^^57^ and the nutrients^37^ they supply in structuring complex communities in the wild. Our work suggests that in coastal settings, for example, high levels of allochthonous nutrients might serve to decouple bacteria from the local primary producers. One important caveat to this idea is the supposition that allochthonous nutrients are more recalcitrant^68^ than those excreted by autotrophs. As a result, an important extension of the work here is to understand how variation in the identity of the exogenously supplied resource impacts the convergence and assembly dynamics observed here. One important limitation of this study is the use of glucose as the exogenously supplied resource. It may be that our results depend on the chemical identity of these nutrients, and in particular on which taxa can and cannot consume those nutrients.

More broadly, our study highlights the need for tractable model systems for studying the role of primary producers in structuring microbial consortia. The marine bacterium Prochlorrococcus has served as a powerful model for understanding the ecology of phototrophic microbes in the ocean.^7^ However, Prochlorrococcus is not to our knowledge genetically tractable and we lack the detailed cell biological insights available in *C. reinhardtii*. In contrast, for *C. reinhardtii*, we lack ecological context and insights.^58^ Our hope is that this study helps take a step toward leveraging the power of model organisms for ecological gain. To that end, utilizing large mutant libraries^40^ in concert with communities like those assembled here could more deeply connect genomic properties with ecological outcomes.

### Limitations of study

This study does not investigate the mechanisms by which the algae interact with the heterotrophs, and therefore we do not know the biological bases of the impact of algae on bacterial communities. We were able to isolate only a limited number of taxa. This restricts the conclusions that can be drawn regarding interactions. We also could not easily dissect bacteria-bacteria interactions. Relative abundance data were used for the analysis. This limits our ability to determine how algae alter the abundances of different bacterial taxa in the community.

## STAR★Methods

### Key resources table

**Table:** 

REAGENT or RESOURCE	SOURCE	IDENTIFIER
**Bacterial and virus strains**		

21 bacterial strains	Isolated from experiments	See Data S8

**Critical commercial assays**		

MiSeq v2 300 cycle	Illumina	MS-102-2002
DNeasy 96 well Blood and Tissue kit	Qiagen	Cat. No. / ID: 69504
DNeasy Power Soil Pro kit	Qiagen	Cat. No. / ID: 47014

**Deposited data**		

Sequence data deposited in Zenodo	Zenodo	Zenodo: https://doi.org/10.5281/zenodo.6760409
Supplemental datasets	Zenodo	Zenodo: https://doi.org/10.5281/zenodo.7682205

**Experimental models: Organisms/strains**		

Chlamydomonas reinhardtii	UTEX culture collection	https://utex.org/products/utex-2244?variant=30991921250394

**Software and algorithms**		

Qiime2	https://docs.qiime2.org/2018.11/tutorials/moving-pictures/	Bolyen et al.^9^
DADA2	https://benjjneb.github.io/dada2/tutorial_1_6.html	Callahan et al.^13^
Biopython	https://biopython.org/	Cock et al.^16^
R - phyloseq	https://github.com/joey711/phyloseq	Lozupone et al.^44^
SILVA ACT	https://www.arb-silva.de/aligner/	Pruesse et al.^53^

**Other**		

Scikit-learn	https://scikit-learn.org/stable/modules/generated/sklearn.decomposition.PCA.html#sklearn.decomposition.PCA	Pedregosa et al.^51^
Matplotlib	https://matplotlib.org/stable/index.html	Hunter^35^
Numpy	https://numpy.org/	Harris^32^
Scipy	https://scipy.org	Virtanen et al.^67^

### Resource availability

#### Lead contact

Further information and requests for resources and reagents should be directed to and will be fulfilled by the lead contact, Seppe Kuehn (seppe.kuehn@gmail.com).

#### Materials availability

This study did not generate new unique reagents.

### Experimental model and subject details

#### Soil samples

Soil samples were collected from a restored prairie (Meadowbrook Park, Urbana, IL) located at $40^{\circ }$ 04’42.9"N and $88^{\circ }$ 12’22.3"W. Sterile scoopulas were used to dig holes about 5 cm deep and soil from the bottom of the holes was collected into separate sterile Falcon tubes for each hole. The samples were then taken to the lab. About 5 g of each soil type was weighed into fresh Falcon tubes. 10 mL double distilled water was added to each Falcon tube. The soil was soft and broke down into fine particles on vortex mixing. This soil suspension was then stored at 4°C.

To extract bacterial communities from the soil samples, the Falcon tube was vortexed thoroughly and the large soil particles were allowed to settle. However, very small soil particles and bacteria remain suspended in the supernatant. This supernatant containing bacteria and small soil particles was collected and centrifuged at 7000 rpm for 5 min. The pellets contained bacteria and small soil particles. The supernatant was discarded and the soil bacterial pellet was re-suspended in media without organic carbon. This was then distributed into sterile test tubes so that each test tube received no more than 2.5 mL total. To each test tube, 200 μg/mL of the drug cycloheximide was added to kill any eukaryotes. Further, 0.02 mg/mL of the fungicide Nystatin was added to kill any fungi. The test tubes were then wrapped in Aluminum foil to prevent light from entering the soil sample, thus prohibiting the growth of any native phototrophs present in the community. These wrapped test tubes were then placed in a shaker-incubator, where they were shaken at 225 rpm and incubated at 30°C for 2 days.

After two days of incubation, the samples were collected from the test tubes and centrifuged at 7000 rpm for 5 min, causing the bacteria and remaining small soil particles to form a pellet. The supernatant, which contained the drugs, was discarded. The bacterial community was then re-suspended in an equal amount of fresh media without organic carbon. The samples were then ready for further use. The absence of eukaryotes was not tested. The communities at this stage will be referred to as initial soil inoculates. 250 μL from two such initial soil inoculates, which are the source of heterotrophs, are used for the experiments, and designated as soil samples A and B.

#### *Chlamydomonas reinhardtii*

We use a lab strain of *C. reinhardtii*, UTEX2244, mt+. A vial of frozen stock is thawed in a water bath at 35°C. The thawed stock is immediately added to an Erlenmeyer flask containing 20 mL Tris-Acetate Phosphate (TAP) media. The culture was grown for three days in continuous light conditions, with shaking at 220 rpm, and at 30°C. After three days’ growth, the culture was centrifuged at 5000 rpm for two minutes. The supernatant was discarded and the cells re-suspended in the 1/2 x Taub media (See Table S1). They were then centrifuged again, at the 5000 rpm for 2 min. Again, the supernatant was replaced with fresh Taub media. This process was repeated again 3 times in total. The cells were then counted using a hemocytometer and the appropriate dilution to get $1\times 10^{5}$ cells/mL initial cell density was computed. To test whether our stock of *C. reinhardtii* was indeed axenic, we streaked the culture on an agar plate made of rich medium and incubated the plate in the dark at 30°C. After three days of incubation, there was no bacterial growth, indicating that the stock was not contaminated.

##### Tris Acetate phosphate (TAP) media

TAP media was used to start the *C. reinhardtii* cultures from the frozen stocks. First, a 2X Filner Beijernicks solution is made by dissolving 8 g NH_4_Cl, 1 g CaCl_2_.2H_2_O and 2 g MgSO_4_.7H_2_O in 500 mL MilliQ water. This stock solution is then autoclaved and stored at 4°C.

Next, a stock of trace mineral solution is prepared. We start with dissolving 5g of disodium EDTA in 400 mL double distilled water by heating and constant stirring. The pH is set to 6.5 using 5N NaOH. Then, the following compounds are added in the given order, ensuring that each compound dissolves before adding the next: FeSO_4_.7H_2_O 0.5 g, ZnSO_4_.7H_2_O 2.2 g, H_3_BO_3_ 1.14 g. MnCl_2_.4H_2_O 0.51 g, CuSO_4_.5H_2_O 0.016 g, Na_2_MoO_4_.2H_2_O 0.073 g, and Co(NO_3_)_2_.6H_2_O 0.0196 g. The volume is then made up to 500 mL and autoclaved.

Next, a phosphate stock is prepared by adding 6.8 g of KH_2_PO_4_ and 8.7 g of K_2_HPO_4_ to 50 ml MilliQ water and autoclaving.

Finally, TAP media is prepared by combining 12.5 mL of the 2X Filner’s Beijernick’s solution, 0.5 mL of the phosphate stock, 2.5 mL of the trace mineral solution, 1.21 g of Tris- base, and 0.5 mL of glacial acetic acid. The volume is brought to 500 mL, the pH adjusted to 7.2, and the solution is autoclaved. It is stored at room temperature.

### Method details

#### Enrichment experiments

The soil inoculates and *C. reinhardtii* were added to glass vials. Two soil samples, A and B, were used. For each soil sample and growth period, there were 5 replicates of hybrid communities and either 3 (soil sample A) or 2 (soil sample B) replicates of the control communities. The vials are made of glass, are cylindrical and have a volume of 40 mL. Each vial contained a stir bar. The vials were closed using sterile foam stoppers, which permitted airflow but maintained sterility. We used large vials, rather than microtiter plates, due to the long timescales of the growth periods where evaporation would cause excessive water loss in smaller volume cultures.

##### Measurement of evaporation

Evaporation was measured by measuring the weight of two vials with water every 24 h in the experimental conditions, i.e, with the foam stopper, with stirring, and with the LED lights on. Subtracting the initial weight of the vial, and using the density of water, the volume of water evaporated was calculated to be about 0.38 mL/day. This means that for the longest growth period of 12 days, the total volume of water evaporated would be 4.5 mL.

We used a modified version of the freshwater mimicking Taub media^62^ to make up the total culture volume of 20 mL. The Taub media (See Table S1) was at pH 7.2 and with defined carbon, nitrogen and phosphate sources. In particular, we note that the amount of glucose we supply (1.33 mM glucose or 8 mM C atoms) is comparable to the amount of carbon secreted by *C. reinhardtii*, which is about 8 mM C atoms over 8 days.^64^

After the communities in the vials have grown for the number of days specified by their growth period, a 1:64 dilution was performed into fresh Taub media. This process was repeated until communities of all growth periods and both soil samples have completed 10 rounds of serial dilution. At the end of each serial dilution, samples were collected to measure Chlorophyll fluorescence and optical density. Samples were also preserved with 25% glycerol at −80 °C and for 16s sequencing at −20 °C. Note that the $\tau_{12}$ and $\tau_{9}$ control communities of soil sample A had 1 and 2 replicates contaminated by *C. reinhardtii* respectively, and these contaminated samples are not used in the analysis.

A multi-position stir plate (2mag-USA) was used to stir 60 vials simultaneously. A multi position vial holder was custom designed to hold 60 vials each of 40 mL volume. An LED light panel (Good Earth Lighting, Item: 805392, Model: LF1089-PEW-28LF4-G) was placed between the vial holder and the multi-position stir plate to provide uniform illumination from below. The light panel was powered by a DC power supply, via a signal relay mechanism. The signal relay was in turn controlled by a Raspberry Pi, which allowed us to turn the lights on and off every 12 h, thus simulating day - night cycles. The vial holder was enclosed in a thick black cardboard box to ensure that external light changes did not affect the experiment. The entire set up was placed in an incubated hot room set to 30°C.

The input voltage (19 V) and current (1 A) to the LED light sheet was set on the DC power supply so that the average light intensity at each position was 1050 lux with a standard deviation of 50 lux across all positions as measured using a luxmeter. This is approximately 30 μmol / m ${}^{2}$/s as measured by an LI-COR light meter. The color temperature of the LED light panel was 3975K (bright white).

#### Optical density and chlorophyll fluorescence measurements

At the end of each round of serial dilution, samples from the communities were transferred to the wells of a 48 well plate. The optical density (OD) was determined by measuring absorbance at 600 nm of the samples in the 48 well plate using a plate reader (BMG Labtech, Clariostar). The OD600 of the Taub media was measured separately, and used as background for background subtraction. The background subtracted OD600 measurements for the hybrid communities are shown in Figure S1A. We note that the OD600 of the $\tau_{12}$ hybrid communities is much higher than that for the $\tau_{3}$ and $\tau_{6}$ hybrid communities by round 10 in both soil samples. In soil A, the $\tau_{9}$ hybrid communities also have a higher OD600 by round 10 compared to the $\tau_{3}$ and $\tau_{6}$ hybrid communities. In comparison, Figure S1B shows the background subtracted OD600 measurements for the control communities. By dilution round 10, control communities in both the soil samples reach a similar OD600 of about 0.3 irrespective of the growth period, despite starting at different OD600 values initially.

Auto-fluorescence of chlorophyll was used to quantify chlorophyll content by measuring fluorescence in a Clariostar Plate Reader at excitation wavelength of 482±20 nm, emission wavelength of 690±20 nm using a dichroic at 585 nm. The results of the measurements for hybrid communities are shown in Figure S1C. In Soil A, the $\tau_{12}$ communities have a higher chlorophyll content. However no other trend is visible.

In order to compare the OD600 of the hybrid communities without the contribution of *C. reinhardtii*, we plotted the ratio of OD600:Chlorophyll fluorescence in Figure S1D. This shows that $\tau_{3}$ hybrid communities have a higher OD:Chlorophyll content compared to hybrid communities of other growth periods for both soil samples. In other words, for the $\tau_{12}$ hybrid communities, which had higher OD600 values, the high OD is a result of the *C. reinhardtii* abundance. Further, the hybrid communities with the shorter growth periods have more bacteria per chlorophyll content than the hybrid communities with the longer growth period. This is consistent with the loss in diversity observed (Figure 3) in the hybrid communities with longer growth period.

#### pH

The approximate pH of $\tau_{12}$ Soil B communities was measured at the end of round 7 of serial dilution using pH paper. We found that the pH of the hybrid communities had dropped to between 5 and 6, whereas the pH of the control communities remained at around 6.4.

Since the pH of hybrid communities dropped a lot more, we hypothesized that *C. reinhardtii* could be responsible. In a media with NH${}_{4}$Cl, algal growth acidifies the media by taking up NH${}_{3}$ and leaving behind H ${}^{+}$ ions.^59^ To test this hypothesis, we grew *C. reinhardtii* in media with two different NH${}_{4}$ Cl concentrations. One had the concentration used in the experiment (8 mM) while the other had a lower concentration of NH${}_{4}$ Cl of 2 mM. In both media, the initial pH was set to 7.2 The pH of the culture was measured every 3 days for 12 days. The drop in pH was steeper in the media with 8 mM NH${}_{4}$Cl than in the media where the concentration was 2 mM. Further by the end of 12 days, the pH had dropped to 5.7 in the media with 8 mM NH${}_{4}$Cl and seemed to be declining with time (negative slope of pH vs. time), whereas in the media with 2 mM NH${}_{4}$Cl, the pH had dropped to 6 and seemed to stabilize there (slope of pH vs. time was about zero).

This indicated that *C. reinhardtti* causes the drop in pH due to the consumption of NH${}_{4}$Cl. The sharper drop in pH seen in the hybrid communities could be due to the contribution of the heterotrophs to the acidification process.

#### DNA extraction

16s amplicon sequencing of the V4 region was performed on all samples from every other round of serial dilution based on the protocol from the Earth Microbiome Project (EMP).^14^ All the primers used were obtained from the sequences provided in the Earth Microbiome Project.^14^

DNA was extracted using Qiagen’s DNeasy 96 well Blood and Tissue kit. A modified version of the protocol was used. Just prior to starting the extraction process, a lysis buffer (see Table S2) was freshly prepared. The samples frozen at -20°C were thawed and centrifuged for 15 min at 21000 rcf. The supernatant was discarded and the pellets were re-suspended in 180 μL lysis buffer. The samples were then incubated for 30 min at 37°C. 25 μL proteinase K and of 200 μL Buffer AL (without ethanol) were added to the samples. The samples were then incubated at 56°C for 1 h. Next, 200 μL ethanol was added to the samples and mixed by vortexing. The samples were then centrifuged briefly (allowed to reach 3000 rpm and then stopped) to collect any solution accumulated in the caps.

A DNeasy 96 well plate was placed on top of an S block (both provided with the kit). The lysate was transferred to the wells of the 96 well plate. The plate was then sealed with an AirPore Tape sheet and centrifuged at 4000 rpm for 15 min. The tape sheet was then removed and 500 μL buffer AW1 was added to all the wells. The plate was then sealed again with a new tape sheet and centrifuged for 10 min at 4000 rpm. After centrifugation, the tape was removed and 500 μL buffer AW2 was added to all samples. The plate was centrifuged for 20 min at 4000 rpm without a tape sheet.

The DNeasy 96 well plate was then placed on a rack of elution microtubes, and 100 μL of elution buffer AE added to all samples. The samples were incubated for 1 min at room temperature and centrifuged at 4000 rpm for 3 min. This step is repeated again. The extracted DNA is in the elution microtube, which is then covered and stored for future use.

#### DNA extraction from soil

The kit used above to extract DNA from liquid cultures was not suitable for extracting DNA from bacteria present in soil. So we used Qiagen’s DNeasy Power Soil Pro kit to extract DNA from the initial soil inoculates (the communities after being extracted from soil and after treatment with drugs for 2 days in the dark) that were used to initiate the experiments, preserved after the treatment with drugs. The beads of the PowerBead Pro tube were removed and the thawed soil sample (preserved in liquid, about 20 mg) was added to the tubes. The tubes were centrifuged at 1000 rcf for 30 s, the supernatant was removed. The beads were then added back into the tube. 800 μL of solution CD1 was added and the tube was vortexed. The tubes were then fixed horizontally into a vortex adaptor and vortexed for 10 min to homogenize the samples. The tubes were then centrifuged at 15000 rcf for 1 min. The supernatant was transferred into micro-centrifuge tubes. 200 μL of solution CD2 was added and the tubes were vortexed for 5 s. Then the tubes were centrifuged at 15000 rcf for 1 min. The supernatant was transferred into a clean micro-centrifuge tube. 600 μL of solution CD3 was then added and the tubes vortexed. 650 μL of the lysate was loaded onto an MB spin column and centrifuges at 15000 rcf for 1 min. The flow through was discarded, and the process was repeated until all the lysate was passed through the column. The MB spin column was then placed in a 2 mL collection tube and 500 μL of solution EA was added to it. After centrifugation at 15000 rcf for 1 min, the flow through was discarded and the spin column placed into the same collection tube. 500 μL of Solution C5 was then added and centrifuged at 15000 rcf for 1 min. The flow through was again discarded the spin column was placed in a fresh collection tube. The tube was then centrifuged at 16000 rcf for 2 min and the spin column was placed in an elution Tube. 75 μL of the Solution C6 was added to the center of the filter membrane and the tube was centrifuged at 15000 rcf for 1 min. The flow through contained the DNA and was stored at -20°C.

#### Library prep

Library prep was performed according to the protocol suggested by Illumina. In particular, first, to ensure that the DNA extraction worked, the DNA concentration was quantified in all samples using Invitrogen’s Qubit dsDNA BR Assay kit, adapted to a 96 well plate version.

#### Qubit

Enough working solution was prepared by mixing Qubit dsDNA BR buffer with Qubit dsDNA BR reagent in a 1:200 ratio. Using the provided standard solutions, one 100 ng/μL and the other 0ng/μL, serial dilutions were performed to obtain a range of known DNA concentrations. 195 μL of the working solution, and 5 μL of the known DNA concentrations were added to the wells of the 96 well plate. The plate was vortxed briefly, allowed to incubate, and placed in a plate reader. The excitation wavelength is 485 nm and the emission wavelength is 530 nm. The wells were scanned using these parameters and calibration curve was obtained.

Once the calibration curve was obtained, the the same procedure described above was used to measure the fluorescence of the unknown samples, using the same working solution. From the calibration curves, the DNA concentration in the samples was inferred.

#### PCR

Once it was confirmed that the DNA extraction process had worked, PCR was performed on the extracted DNA in accordance with the Earth Microbiome protocol.^14^ All samples received the same forward primer but unique reverse primers. Each reverse primer has a unique barcode, which can be later used to demultiplex reads belonging to each community. The reaction were performed in triplicate (see Table S3 for the volumes) with a total volume of 25 μL each. The master mix used was the Platinum Hot Start Master mix. See Table S4 for thermocycler settings.

After PCR was performed on all samples, the triplicates were pooled. The DNA concentration of each sample was measured using the Qubit dsDNA BR Assay kit as described above. For each sample, the volume required to obtain 240 ng of DNA was calculated. This volume was then collected from the respective samples and pooled. The total volume of the pool was also calculated.

The pooled PCR products were then cleaned using QIAquick PCR purification kit. Ethanol was added to buffer PE prior to use. pH indicator was added to the buffer PB in a ratio of 1:250. Yellow color of the resulting mixture indicated a pH >7.5. 5 volumes of the buffer PB were added to the 1 volume of the PCR products and mixed. A QIAquick spin column was placed in a collection tube. The sample was then added to the spin column and centrifuged at 17900 rcf for 45 s. The flow through was discarded and the spin column was placed back in the same collection tube. 750 μL of the buffer PE was added to the spin column and centrifuged at 17900 rcf for 45 s. The flowthrough was discarded and the spin column was placed back in the same tube and centrifuged at 17900 rcf for 1 min. Then the spin column was placed in a clean microcentrifuge tube. 30 μL of the buffer EB was added to the center of the spin column’s membrane and let stand for a minute. The column was then centrifuged at 17900 rcf for 1 min. The flow through contained the cleaned and pooled PCR products.

The final concentration of the cleaned and pooled PCR products was measured using the Qubit dsDNA BR Assay kit as described above. The concentration of the DNA was estimated using the following formula^36^:

(Equation 1) $$\frac{\text{concentration}\;\text{in}\;\text{ng}/\mu L}{660\;g/\text{mol}\times \text{average}\;\text{library}\;\text{size}}\times 10^{6}=\text{concentration}\;\text{in}\;\text{nM}$$

For our experiments, the average library size was 390. Using resuspension buffer, the library was diluted to a concentration of 4 nM.

#### Sequencing

A MiSeq Illumina machine was used with a v2 300 cycle kit. The sequencing was carried out in accordance with. Fresh 0.2 N NaOH was prepared and used to dilute the 4 nM pooled library to 2 nM. It was then vortexed and incubated at room temperature to 5 min to allow denaturation. The denatured DNA was then diluted to 20 pM using pre-chilled HT1 buffer and then further to 8 pM using the same pre-chilled HT1 buffer.

Next, a PhiX control was diluted to 4 nM using resuspension buffer. Using the freshly prepared NaOH, PhiX was diluted down to 2 nM, which was then vortexed and incubated at room temperature to allow denaturation. The, using pre-chilled HT1 buffer, the library was diluted to a 20 pM library, and them further to 8 pM.

Finally, the denatured PhiX and the denatured sample library were pooled so that PhiX made up 5% of the mixture. The mixture was then placed in a heatblock at 96°C for 2 min.

A .csv file was prepared with the information about the samples and the kit details. This file was loaded onto the MiSeq machine. The thawed cartridge was flipped up and down a few times. The foil marked for sample was pierced using a pipette tip and the sample loaded into it. Reservoirs 12, 13 and 14 were also pierced with clean pipette tips. 3.4 μL of 100 μM Index sequencing primer was added to reservoir 13, 3.4 μL of read 1 sequencing primer was added to reservoir 12, and 3.4 μL of read 2 sequencing primer was added to reservoir 14. The kit was then loaded into the machine, and the run was started.

#### Isolation of strains

To isolate individual strains from the communities, the stock preserved in glycerol was used. These stocks were grown in vials in Taub media in the presence of *C. reinhardtii* at the same concentration as in the enrichment experiments. The communities were allowed to grow for the same number of days as in the experiment with cycles of light and dark i.e., a 3 day community was re-grown for 3 days, a 9 day community for 9 days and so on. At the end of the growth, the communities were diluted and plated on R2 agar plates and Taub agar plates.

When individual colonies grew, each distinct looking colony was selected and grown on a fresh plate. This process was repeated until it was clear visually that the the strains were pure.

Colony PCR (see Table S5 for volumes and Table S6 for thermocycler settings) was performed on these strains by picking a single colony and dissolving it in 100 μL PCR grade water. The same forward primer and one of the reverse primers used in the EMP protocol were used. The master mix used for the EMP protocol was also used here.The PCR products were sent for Sanger sequencing. The results of the Sanger sequencing were compared with the results of Illumina MiSeq sequencing to assign the right label to each isolated strain. To do this, we used Biopython^16^ SeqIO’s “abi-trim" option, which trimmed the input Sagner sequence based on the quality scores using Mott’s algorithm. To align the Sanger sequence with the 16s MiSeq reads, we used Biopython’s local alignment, with a match score being the score of identical characters, and with penalties for opening and extending gaps. In particular, identical characters were given a score of 4, 2 points were deducted for every non-identical character, 2 points were deducted for opening a gap and 1 point was deduced for extending an open gap.

Through this process, 21 unique bacterial isolates were obtained. See Data S8 for more details of the isolated taxa.

#### Carbon utilization assays

Individual isolates were grown in 2 mL R2 media in test tubes at 30 °C with constant shaking for 48h. After 48 h, 1 mL of the cell cultures were centrifuged at 21300 rcf for 2 min. The cells were washed three times in the Taub media. Then, the optical density of cell cultures (absorbance at 600 nm) was measured. The volume required to have a starting OD of 0.01 in a 750 μL solution was calculated for each isolate to get three replicate communities. In a 48 well plate, the calculated volumes of the cell cultures were added into 3 wells per isolate. The rest of the 750 μL volume was made up by the Taub media. The plate was then sealed with parafilm and the placed in the plate reader with incubation at 30°C. Absorbance at 600 nm was then measured continuously every 10 min for about 4 days. In between the measurements, the plate underwent orbital shaking at 400 rpm.

Seven different sources of organic carbon were used in the Taub media. One was glucose, and the other 6 were chosen from the metabolomics data of the algal spent media (STAR Methods "chemical analysis of algal exudates"). The growth curves of isolates on all 7 of these carbon sources were measured using the method described above.

The growth rate and maximum OD reached were calculated from the growth curves by plotting the data on a semi-log scale, after background subtraction. A small number was added to all the data to make all the points positive. The curves were then fit using a spline. The maximum yield (maximum OD600) was found, and the maximum growth rate was inferred from the first derivative of the spline fit. The maximum growth rates of all the isolates in the 7 carbon sources tested is showin in Figure S19.

To binarize the data for the LASSO regression analysis (see below), any isolate with a maximum OD600 of 0.05 or higher on a particular carbon source was considered to have utilized that carbon source. If the isolate did not reach an OD600 of 0.05, it was considered to be incapable of growing on that carbon source.

#### Co-culture experiments via flow cytometry

To study the impact of bacteria on the growth of *C. reinhardtii* we grew *C. reinhardtii* on it’s own, and in co-culture with individual bacterial strains. Bacterial isolates were first grown in R2 media for two days, then diluted into the Taub minimal media. After a further two days of growth, the bacterial isolates were inoculated at a starting OD of 0.0005. Bacterial isolates that did not grow well in liquid media were directly plated and the colonies were used for the experiments. As in the enrichment experiments, *C. reinhardtii* was grown in TAP media and washed three times in the Taub media on saturation. It was then diluted to start the experiments at a low cell density of 100 cells/mL in order to observe the growth curves. Each condition had 3 replicates. The cultures were grown at the same temperature, stirring and light conditions as the enrichment experiment. Samples were collected regularly for measurement of *C. reinhardtii* cell densities. These samples were fixed using the drug cycloheximide (100 μg / mL), and stored at 4°C. After a number of samples were collected, they were transferred to a 96 well plate and the cell density was measured using the Attune Nxt 14 flow cytometer housed at the Cytometry and Antibody Technology Core facility at the University of Chicago (Voltages: Forward scatter: 360 V, Side scatter: 200 V, Laser: 260 V). It is a volumetric flow cytometer that can measure flow volume, eliminating the need to use beads for calibration. This resulted in a time series of algal abundances in mono-culture and in co-culture with bacterial isolates. The raw data are shown in Figure S15.

To assess the impact of bacteria on algal growth we computed the area under the abundance verses time curve (AUC)^31^ acquired via flow cytometry. The area under the growth curve decreases as either the lag time increase, growth rate decreases, or carrying capacity falls. Therefore, any reduction in the AUC in the presence of bacteria relative to the algae alone indicates an interaction. If the AUC increases this suggests bacterial facilitation of algal growth and if it decreases this suggests inhibition. Therefore, we computed the ratio of the AUC (area with isolate/area without isolate). If this ratio is larger than 1, then that isolate is beneficial for the growth of the algae, and vice versa.

#### Co-culture experiments via plating

To study the effect of *C. reinhardtii* on isolates, each isolate was grown on its own and in the presence of *C. reinhardtii*. Bacterial isolates were first grown in R2 media for two days, then diluted into the Taub minimal media (including glucose, as used in the enrichment experiments). After a further two days of growth, the bacterial isolates were inoculated at a starting OD of 0.001. Bacterial isolates that did not grow well in liquid media were directly plated and the colonies were used for the experiments. As in the enrichment experiments, *C. reinhardtii* was grown in TAP media and washed three times in the Taub media on saturation. It was then diluted to start the experiments at a cell density of $10^{5}$ cells/mL. After 14 days of growth in the same conditions as the enrichment experiment, the communities were serially diluted and 10 μL of the serially diluted cultures were plated on agar plates^65^ to measure the end point cell density of the isolates when grown on their own and when grown in the presence of algae. The lowest dilution factor used was $10^{-4}$. Due to this, the detection limit of the cell density in this method is $1\times 10^{6}$ cells/mL.

#### Chemical analysis of algal exudates

To find the organic carbon compounds excreted by *C. reinhardtii*, we grew the algae axenically in three replicates (without any soil communities, or other bacteria). Samples were collected at the end of 3 days, 6 days, 9 days and 12 days, corresponding to the growth periods used in the experiment. The samples were then sent for analysis using gas chromatography - mass spectrometry (GC-MS) to the Roy Carver biotechnology center at the University of Illinois at Urbana Champaign (Agilent 7890A GC/5975C MS). Samples were derivatized with 100 μl of methoxyamine hydrochloride (40 mg /mL in pyridine) for 90 min at 50°C and then with 100 μL MSTFA at 50°C for 120 min. 20 μL of the internal standard (hentriacontanoic acid, 1 mg /ml) was added to each sample prior to derivatization. Samples were analyzed on a GC/MS system (Agilent Inc, Palo Alto, CA, USA) consisting of an Agilent 7890 gas chromatograph, an Agilent 5975 mass selective detector, and a HP 7683B autosampler. Gas chromatography was performed on a ZB-5MS (60 m × 0.32 mm I.D. and 0.25 μm film thickness) capillary column (Phenomenex, CA, USA). The inlet and MS interface temperatures were 250°C, and the ion source temperature was adjusted to 230°C. An aliquot of 1 μL was injected with the split ratio of 10:1. The helium carrier gas was kept at a constant flow rate of 2.4 mL/min. The temperature program was: 5 min isothermal heating at 70°C, followed by an oven temperature increase of 5°C/min to 310°C and a final 10 min at 310°C. The mass spectrometer was operated in positive electron impact mode (EI) at 69.9 eV ionization energy in m/z 30-800 scan range. All known artificial peaks were identified and removed. MS peaks were evaluated by AMDIS 2.71 (NIST, Gaithersburg, MD, USA) program and metabolites were identified by custom-built EI-MS library (484 unique metabolites). To allow comparison between samples, all data were normalized to the internal standard. The resulting data indicated the presence of about 40 different organic compounds (Data S7). To infer which of these compounds were being secreted increasingly with time, we subtracted the baseline compounds found in the media, and then plotted the measurements as a function of time. We then performed a linear regression, and found which compounds had a significant positive slope. The data from GC-MS are provided in the Data S7 and the compounds that are secreted in significant amounts are listed in Table S7.

### Quantification and statistical analysis

For majority of the quantification and analysis, Python was used, in particular, Scipy^67^ and Numpy.^32^ Matplotlib^35^ was used for generating the figures.

#### 16s sequence data processing

Fastq files were generated on Illumina’s MiSeq sequencing machine after the run was completed. This gave 3 files, one each for the forward, reverse and barcode reads.The fastq files were then analyzed downstream using Qiime2^9^ and DADA2.^13^ The fastq files are de-multiplexed in Qiime2 using a file assigning barcodes to sample names. The reads were then passed through a quality control check and phiX reads were filtered.

##### Amplicon sequence variants (ASVs)

In order to obtain ASVs, the de-multiplexed reads were analyzed using DADA2 in R using the DADA2 pipeline.^12^ The quality of the reads, both forward and reverse, were plotted and it was found that due to the good quality of reads, trimming was not necessary. The reads were then filtered for the number of expected errors. The error rates were learnt from the dataset for the forward and reverse reads. Next, dereplication was performed on the forward and reverse reads to combine the identical reads into unique sequences, with counts information. Using the error rates calculated above, the number of unique sequences were inferred from the dereplicated reads. Next, the forward and reverse reads were merged, a sequence table constructed, and chimeras removed. Finally, taxonomy was assigned by using SILVA’s reference database (v128). The ASV level composition of the initial soil inoculates is shown in Figure S2 and for the communities is shown in Figure S3. See Data S1 for the counts data and Data S2 for the phylogeny data at the ASV level. The ASVs with the same Genus were grouped to find the community composition at the Genus level. If the Genus was not assigned, the next higher taxonomic classification was used, and the ASV number appended to distinguish the different groups. This is shown in Figure S4.

##### Operational taxonomic units (OTUs)

In order to obtain OTUs, the de-multiplexed files were denoised in Qiime2 using the DADA2 plugin.^54^ Then, using Qiime2’s vsearch option, the reads were clustered at 97.5% similarity to obtain the count table for the OTUs. This counts table was exported for further analysis. The taxonomy was assigned using Greengenes database on Qiime2. See Data S3 for the counts data and Data S4 for the phylogeny data at the OTU level. The fasta file for these data can be accessed at Zenodo Data: https://doi.org/10.5281/zenodo.6760409.

#### Shannon diversity

Shannon diversity was calculated for each community on all the data i.e., without removing rare taxa. This diversity index is calculated as:

(Equation 2) $$H=-\sum_{i}p_{i}log_{e}p_{i}$$

Here H is the Shannon diversity index of a community, $p_{i}$ is the probability of finding taxa *i* in a given community (relative abundance), and the summation is over all the taxa.

#### Distance metrics

The counts per taxa was converted to relative abundance for each community by dividing the number of counts for each taxa by the total number of counts for that community. To compare the similarities and differences between the communities, several metrics were used. They are described here.

##### Jensen-Shannon divergence

Given that the data are in the form of relative abundances, each community can be considered as a normalized probability distribution of the taxa. In this case, Jensen-Shannon divergence (JSD) is a good metric to be used,^41^ because it is bounded, has the capability to be weighted and is symmetric.^41^ The JSD between two normalized probability distributions, *X* and *Y*, is given by Equation 3, where H is the Shannon entropy of the probability distribution and $\pi_{1}$ and $\pi_{2}$ are the weights for the two distributions.

(Equation 3) $$J_{X,Y}=H\left(\pi_{1}X+\pi_{2}Y\right)-\pi_{1}H\left(X\right)-\pi_{2}H\left(Y\right)$$

If the distribution *X* is given by $X=\left\{x_{i}\right\}$, where $x_{i}$ represent the normalized probability of finding the value $x_{i}$ in the probability distribution *X*, the entropy *H* of the probability distribution *X* is defined by Equation 4.

(Equation 4) $$H\left(X\right)=-\sum x_{i}\;\log\;x_{i}$$

Here, we set the weights to be $\pi_{1}=\pi_{2}=\frac{1}{2}$. Using Equation 3, we can now define the Jensen Shannon divergence between the relative abundance composition of the different communities.

In order to assess if the hybrid communities are indeed different from control communities, we found the JSD between all pairs of hybrid and control communities. If $H_{i,g}^{r,s}$ is the $i^{th}$ replicate of a hybrid community of soil sample *s*, with growth period *g* and at dilution round *r*, and $C_{j,g}^{r,s}$ is the $j^{th}$ replicate of a control community of soil sample *s*, with growth period *g* and at dilution round *r*, we first found the set of distances $H_{i,g}^{r.s}$-$C_{j,g}^{r,s}$, for all pairs i,j. We term this the inter community distances. Next, as a comparison, we found the distances within each hybrid community of a given growth period and dilution round, i.e. we found the set of distances $H_{i,g}^{r,s}$-$H_{j,g}^{r,s}$ where i,j∈{1,2,3,4,5} and i≠j. We term these the intra hybrid community distances. We now have a distribution of inter community distances and intra hybrid community distances. We used bootstrapping (sampling with replacement) to generate different instances of these two distributions. At each instance, we find the difference in medians of the two distributions, *d*. So, at the end, we have a distribution of difference in these medians. Figure S5 shows the distribution of these difference in medians for the each soil sample, growth period, and dilution round. We observe that *d* is larger than zero (p value <0.002, with the null hypothesis that *d* is equal to or less than zero) for the $\tau_{12}$ and $\tau_{9}$ communities in both soil samples by dilution round 10, whereas for $\tau_{3}$ communities *d* is close to zero even by dilution round 10 (p value 0.13). This means that the hybrid communities are much more distinct from the control communities for the 12 and 9 days growth periods than the 3 day growth periods. This shows that the impact of *C. reinhardtii* on community assembly is stronger for the longer growth periods where the supply of external nutrients is infrequent.

##### Aitchison’s distance metric

Aitchison’s distance metric is a way to measure taxonomic distances between communities when the data are compositional, and has some advantages over Bray - Curtis and Jensen Shannon distance metric that are discussed elsewhere.^24^

The data are first clr transformed, and then the Euclidean distance is found between all community pairs. The distances are then embedded on 2 dimensions using Multi-dimensional embedding (Figure S9 top panel). The stress of the multi-dimensional embedding is shown in the bottom panel of Figure S9.

The embedding of the Aitchison distance shows similar results as the PCA analysis, with the hybrid communities of the longer growth periods converging to become taxonomically similar, while the other communities do not.

##### Unifrac

The distance measures in PCA or the Aitchison analysis above do not account for the phylogenetics of the taxa. We wanted to check whether our conclusions were robust to including phylogenetic relationships. To account for the phylogenetic information in finding the distances, we used the unweighted Unifrac distance metric. For this, a tree file was first created by uploading the fasta file of the sequences to SILVA’s Alignment, Classification and Tree Service.^53^ The tree, along with the counts matrix was then used as input for R’s phyloseq package to create a phyloseq object. The newick treefile is available as Data S9. Using this object, the unweighted Unifrac distances were computed on R^44^ between all pairs of samples. These distances were then embedded in 2 dimensions using multi-dimensional scaling. The results are shown in panel A of Figure S10 and the stress of the embedding is shown in panel B.

The embedding of the Unifrac distance shows similar results as the PCA analysis, with the hybrid communities of the longer growth periods converging to become taxonomically similar, while the other communities do not.

#### Principal component analysis on the taxonomic data

PCA was performed on the 16S amplicon sequencing data. However, the data are compositional and need to be analyzed accordingly.^24^^,^^25^ A standard way to do this is to transform them using a log transformation. But to perform log-transformations, the data cannot have zeros. But sequence data have typically have a large number of zeros. Here we describe how we handle these problems.

##### Aitchison’s transform

Here, we use the center-log-ratio (clr) transformation, suggested by Aitchison.^1^^,^^2^ If $\vec{x}$ is the vector of the composition of each community, and $x_{i}$ are the counts of each individual taxa *i*, with i∈(1,2,…N) and *N* the total number of OTUs found across all communities, then the clr transformation is:

(Equation 5) $$\text{clr}\left(\vec{x}\right)=\left[\ln\frac{x_{1}}{g\left(\vec{x}\right)},\ln\frac{x_{2}}{g\left(\vec{x}\right)},\ln\frac{x_{3}}{g\left(\vec{x}\right)},\ldots ,\ln\frac{x_{N}}{g\left(\vec{x}\right)}\right]$$

where $g\left(\vec{x}\right)=\sqrt[{N}]{x_{1}\cdot x_{2}\cdot x_{3}\cdots x_{N}}$ is the geometric mean.

##### Zero removal

From above, it is clear that zero counts will cause the geometric mean to be zero and the transformation will therefore diverge. Although many methods have been suggested to replace zeros,^50^ they are not always suitable for all datasets.^45^ Here, we explain our method of handling the zeros.

First, we add a small number (1) to all counts. It has been shown elsewhere^61^ that adding small pseudocounts does not affect the downstream analysis process, while effectively removing any zero counts. Then, we clr transform the data, and perform PCA on it. We plot the distribution of the eigenvalues (see Figure S6A). We note that there are 2 eigenvalues that are much higher than the others. Next, we randomly scramble the data, i.e. for each community, we randomly interchange the counts for each taxa (shuffle columns of the data matrix). We clr transform this randomized data and perform PCA on it. This scrambled data does not have any structure, and we expect the high eigenvalues to vanish. The distribution of eigenvalues is shown in Figure S6B. Indeed, we note that the top two eigenvalues disappear.

To remove rare taxa, we plotted the distribution of the frequency of the relative abundances as a function of the maximum relative abundances across all samples at all time points. We observe from Figure S7 that there are a large number of taxa that have a low maximum relative abundance across all samples at all times - which means these taxa have close to zero counts in all communities. We set a cutoff at a threshold of -8.2 for the logarithm of the maximum relative abundance, which corresponds to a relative abundance of 0.00027. All taxa that have a logarithm of maximum relative abundance below this number are discarded. With this reduced data, we re-do the above analysis, i.e., we add a small number, 1, to all the counts, take the clr transform and perform PCA, using sckit-learn.^51^ We plot the eigenvalue distribution for this data in Figure S8A. Again, we note that there are only 2 eigenvalues that are much higher than the others. Next, as before, we randomize the data, clr transform it, and perform PCA. The eigenvalue distribution for this dataset shows that both the high eigenvalues vanish as expected (Figure S8B). This disappearance of the two high eigenvalues on randomizing leads us to conclude that the data are well described by 2 eigenmodes. For the rest of the analysis, we use the reduced data, with the extremely rare taxa discarded.

#### PCA without removing rare taxa

For completeness, we performed PCA on the data without removing rare taxa. The results are shown in Figure S6C, plotted similar to Figure 6. As in the case where the rare taxa were removed, $\tau_{9}$ and $\tau_{12}$ hybrid communities converge, with communities of Soil sample B moving downwards along PC2. The control communities and the $\tau_{3}$ and $\tau_{6}$ communities do not converge. All the communities, irrespective of growth period and presence of algae move along PC1.

#### Displacement along PC2

In order to find the taxa responsible for the displacement of the $\tau_{12}$ and $\tau_{9}$ hybrid communities of soil sample B, we first note that the eigenvector along PC2 is a vector of loadings along PC2 corresponding to each taxon *k*, i.e., $P\vec{C}2=\left\{p_{1}^{2},p_{2}^{2},p_{3}^{2},\ldots ,p_{k}^{2},\ldots \right\}$, where $p_{k}^{2}$ is the loading corresponding to taxon *k* along PC2 (denoted by the superscript 2). The composition of a community can also be represented as a vector of the abundances of the taxa in it, i.e., $\vec{c}=\left\{x^{1},x^{2},x^{3},\ldots ,x^{k},\ldots \right\}$, where $\vec{c}$ represents a community and $x^{k}$ is the mean subtracted, clr transformed abundance corresponding to taxon *k*. The displacement of a community along PC2 is actually a summation over all the taxa, i.e. $d_{\tau }=\left|\left\langle {\vec{c}}_{B,2}^{\tau }|P\vec{C}2\right\rangle -\left\langle {\vec{c}}_{B,10}^{\tau }|P\vec{C}2\right\rangle \right|=\sum_{k}\left|\left(x_{B,2}^{k,\tau }-x_{B,10}^{k,\tau }\right)\right|p_{k}^{2}$, where ${\vec{c}}_{B,r}^{\tau }$ represents the hybrid community at round r, soil sample B, with growth period τ (here, either 9 or 12 days). Here, the notation $\left\langle \vec{p}|\vec{q}\right\rangle$ denotes the inner product of the two vectors $\vec{p}$ and $\vec{q}$ . The summation is over all the taxa *k* and $p_{k}^{2}$ is the eigenvector loading corresponding to taxon *k* along $P\vec{C}2$, and $x_{B,r}^{k,\tau }$ is the mean subtracted, clr transformed abundance corresponding to taxon *k* at round *r*, of soil sample B, with growth period τ. With this we can find the contribution of each taxa to the displacement of the corresponding communities. The contribution of taxa *k* to the displacement of a community of soil sample B along PC2 is $o_{k}=\left|\left(x_{B,2}^{k,\tau }-x_{B,10}^{k,\tau }\right)\right|p_{k}^{2}$.

We computed $o_{k}$ for all taxa *k* for each replicate community. The median across the replicates was used as the contribution of the taxa to displacement along PC2 for that community. The cumulative distribution was obtained for all taxa for each growth period separately, and a cut-off value was chosen to be at 97.5 percentile. The taxa that had a $o_{k}$ higher than this cutoff were retained as ‘algae-driven’ taxa.

We performed this analysis on the dataset with all the taxa (Figures S6D and S6E) and on the dataset after removing rare taxa (Figure 5), obtaining similar results.

##### Average net relative enrichment of algae-driven taxa

For a given OTU $s\in S_{\tau_{12}}$ or $s\in S_{\tau_{9}}$, we found $\Delta r_{h,k,s}^{i,i+1}=r_{h,k,s}^{i+1}-r_{h,k,s}^{i}$. Here, *h* indicates a hybrid community, *i* represents the round of dilution and *k* the replicate. We averaged over all time points and replicates (Figure 5C) to find the average net enrichment in the presence of the algae, $r_{h,s}={\left\langle \Delta r_{h,k,s}^{i,i+1}\right\rangle }_{i,k}$. Note that the sign of $r_{h,s}$ tells us, on average, whether the relative abundance of OTU *s* is increasing or decreasing over dilution rounds.

#### Displacement along PC1

The contribution of each taxa to the displacement along PC1 is found in a similar manner. As before, the eigenvector along PC1 is a vector of loadings along PC1 corresponding to each taxon *k*, i.e., $P\vec{C}1=\left\{p_{1}^{1},p_{2}^{1},p_{3}^{1},\ldots ,p_{k}^{1},\ldots \right\}$, where $p_{k}^{1}$ is the loading corresponding to taxon *k* along PC1 (denoted by the superscript 1). The composition of a community can also be represented as a vector of the abundances of the taxa in it, i.e., $\vec{c}=\left\{x^{1},x^{2},x^{3},\ldots ,x^{k},\ldots \right\}$, where $\vec{c}$ represents a community and $x^{k}$ is the mean subtracted, clr transformed abundance corresponding to taxon *k*. Here, we find the contribution of the taxa to the displacement along PC1 for all 4 growth periods, for both hybrid and control communities. The displacement along PC1 is given by $d_{\tau }=\left|\left\langle {\vec{c}}_{s,2}^{\tau }|P\vec{C}1\right\rangle -\left\langle {\vec{c}}_{s,10}^{\tau }|P\vec{C}1\right\rangle \right|=\sum_{k}\left|\left(x_{s,2}^{k,\tau }-x_{s,10}^{k,\tau }\right)\right|p_{k}^{1}$, where ${\vec{c}}_{s,r}^{\tau }$ represents the community at round r, soil sample *s*, with growth period τ. The summation is over all the taxa *k* and $p_{k}^{1}$ is the eigenvector loading corresponding to taxon *k* along PC1, and $x_{s,r}^{k,\tau }$ is the mean subtracted, clr transformed abundance data corresponding to taxa *k* at round *r*, of soil sample *s*, with growth period τ.

With this we can find the contribution of each taxa to the displacement of the corresponding communities. The contribution of taxon *k* to the displacement of a community of soil sample *s*, growth period τ along PC1 is $o_{k}=\left|\left(x_{s,2}^{k,\tau }-x_{s,10}^{k,\tau }\right)\right|p_{k}^{1}$.

Using the eignevector loadings along PC1, we find $o_{k}$ for all taxa *k* for each replicate community. The median value of $o_{k}$ across the replicates was assigned as the contribution of that taxa for that community’s displacement along PC1. The cumulative distribution of the contributions of all taxa for each growth period was obtained and a cut-off value chosen to be at 97.5% percentile. The taxa that have a $o_{k}$ higher than this cutoff were retained as ‘dilution-frequency driven’ taxa.

In order to test whether the dilution-frequency driven taxa selected depend on the presence or absence of *C. reinhardtii*, we performed Fisher’s exact test^22^ on the selected taxa. The null hypothesis was that the selected taxa are equally likely to belong to the hybrid and the control communities. We found the p-value for each selected OTU. There were 77 OTUs selected in total for the thresholded data. To account for the multiple testing, we used the Bonferroni correction^47^ to obtain a corrected cutoff for the p-value to be 0.000649. None of the selected taxa had a p-value lower than this in either soil sample (Data S5). This implied that the null hypothesis could not be refuted, which means that the selected taxa had an equal probability to be selected from either the hybrid or the control communities. This result means that those taxa that contribute to motion along PC1 are not specific to hybrid or control communities and we therefore refer to them as dilution-frequency driven taxa.

Similarly, we performed this analysis on the entire dataset without removing the rare taxa. For this, our method selected 721 OTUs as dilution-frequency driven taxa. The Bonferroni-corrected p-value for this set is $6.94\times 10^{-5}$ instead of 0.05. The p-values are in Data S6. Out of 721 OTUs, only 2 OTUs have p-value lower than the Bonferroni corrected p-value - OTU2709 (Order - Sphingobacteriales) and OTU2528 (Genus - Novosphingobium).

#### Predicting enrichment rates from carbon catabolic phenotypes

To test if the carbon consumption capability can predict the enrichment rates of the bacterial isolates (Figure S17A), we regressed the enrichment rates on the carbon consumption capability. The carbon consumption ability was obtained from the growth measurements - whether or not an isolated strain could grow on a carbon source. As described earlier, maximum OD600 was used to obtain the maximum yield of the isolates on the different carbon sources. Strains that reached a maximum OD600 of 0.05 or beyond on a carbon source were designated as able to consume that carbon source; those that did not reach an OD600 of 0.05 on a given carbon source were designated as being unable to grow on that carbon source. The growth data were binarized in this way for the regression. This is the binary matrix in Figure S17A.

We have 21 isolates and 7 carbon sources. To prevent over-fitting, we used LASSO regularized linear regression to fit the model given by

(Equation 6) $$r_{h,s}=\beta_{0}+\sum_{j=1}^{7}\beta_{j}g_{j,s}+\epsilon_{s}$$

where $g_{j,s}\in \left\{0,1\right\}$ represents the carbon utilization capability of isolate *s* for carbon source *j*, $\beta_{j}$ are the regression coefficients for the carbon source *j*, $\beta_{0}$ is the intercept, and ${\hat{r}}_{h,s}$ represents the enrichment rate predicted by the LASSO regression, and $\varepsilon_{s}$ is a noise term. The LASSO regression imposes a penalty on the regression coefficients (Figure S17B) $\beta_{j}$ by using the cost function to $\frac{1}{N}∥r_{h,s}-{\hat{r}}_{h,s}{∥}_{2}^{2}+\lambda ∥\beta {∥}_{1}$, where N is the number of samples, here the number of isolates and is equal to 21, and λ is the regularization hyperparameter. The algorithm first optimizes λ to find the lowest mean squared error in the predictions. This is done by iterated cross validation. The data are split into 4-folds. Holding 1-fold out, the regression is fit to the rest of the data for a range of λ values. The coefficients obtained from the fit are used to predict the held out data, and the mean squared error is computed between the held out data and the prediction for each λ. This process is repeated by holding out each of the folds. This process is repeated 100 times, each time by splitting the data into different sets of 4-folds. This gives the mean squared error for the range of λ values used. From this, the λ corresponding to the minimum mean squared error is used as the optimal hyperparameter $\hat{\lambda }$. If the LASSO regression is able to find a sparse set of regression coefficients that predict the response variable well, there is a clear minimum in the mean squared error for a particular λ as seen in the left hand column of Figure S16.^23^ If the regression fails, no clear minimum is obtained as seen in the right hand column of Figure S16.

We find that the regression is successful for the $\tau_{12}$ hybrid communities of Soil A and the $\tau_{12}$ and $\tau_{9}$ hybrid communities of Soil B, but fails for all others. For the control communities, naturally, $r_{c,s}$ is used as the enrichment rate instead of $r_{h,s}$. This indicates that when the algal impact on community assembly is strong, the carbon consumption capabilities of a bacterium can predict its enrichment rates, but in the control communities and the $\tau_{3}$ and $\tau_{6}$ hybrid communities where *C. reinhardtii* has little impact on community assembly, a strain’s carbon consumption capabilities has no predictive power on the enrichment rates.

For each regression, LASSO estimates the regression coefficients $\beta_{j}$ which are the importance of each carbon source for predicting enrichment rates. For the cases where the regression is successful, we plot the regression coefficients corresponding to each carbon source in the top panels of Figures S17C–S17E. For Soil B, Maltose, Ribose and Pyroglutamic acid have predictive power on the enrichment rates, and in Soil A, Maltose and Pyroglutamic acid have predictive power on the enrichment rates. It is noteworthy that glucose is not picked up as having predictive power in either of these cases. This result suggests that growth on carbon excreted by the algae is predictive of enrichment rates in the long growth period conditions.

To assess the out-of-sample predictive power of the model, we divided the data into test sets and training sets in numerous different combinations (4- fold split with 100 repeats). At the optimized penalty coefficient (hyperparameter), $\hat{\lambda }$, we fit the regression on the training set, obtain the regression coefficients and predict the test set. For each of these predictions, we obtained an $R^{2}$ score which determines the goodness of fit. The distribution of $R^{2}$ determined by cross-validation are plotted in the first column of the bottom panels of Figures S17C–S17E. To assess the significance of these values, we performed the regression again on synthetic data where the response variable (enrichment rates) were shuffled (randomized). For the shuffled data, we found the best penalty parameter $\hat{\lambda }$, and repeated the process just described to get the $R^{2}$ scores. We repeated this for 10 different shuffles, each with 4 fold splits and 10 repeats, to get a distribution of $R^{2}$ scores. This is plotted in the second column of the bottom panels of Figures S17C–S17E.

To compare the two distributions, we performed bootstrapping. We sampled the two distributions with replacement numerous times. Each time, we found the medians of the resulting distributions and subtracted them. This gave us a distribution of these differences. We tested for the null hypothesis that the median of the shuffled data is the same as or greater than the median of the actual data. In all cases, we found p-value <0.0001, refuting the null hypothesis. This means that statistically, the regression on the actual data had significant predictive power compared to the null.

## Data Availability

• Sequence data (16s amplicon sequencing) and other datasets have been deposited at Zenodo and are publicly available as of the date of publication. Accession numbers are listed in the key resources table. • This paper does not report original code. • Any additional information required to reanalyze the data reported in this paper is available from the lead contact upon request.

## Supplemental information

Document S1. Figures S1–S19 and Tables S1–S7

Data S1. Abundance data at ASV level Related to STAR Methods and Results section “algal impact on taxonomic composition depends on dilution frequency” and Figures S2 and S3. Here the 16S sequence counts for all the samples are presented. The rows represent the samples and columns represent the ASVs. The samples are named as follows: Soil Sample (A or B) Replicate (r1, r2, etc.) Community type (h for control communities and hcr for hybrid communities) Growth Period (3days, 6days, 9days, or 12days) Serial Dilution Round (r2, r4, r6, r8, or r10). So, a sample with the name “Ar3hcr6dr6″ represents soil sample A, replicate 3, hybrid community, 6 days growth period, and dilution round 6. The name "CR only" followed by a number indicates a sample with only *C. reinhardtii*. The numbers following the name represent the replicates. Cultures of only *C. reinhardtii* were sequenced to identify their contribution to the 16S sequence data.

Data S2. Phylogeny of ASV data Related to STAR Methods and Results section “algal impact on taxonomic composition depends on dilution frequency” and Figures S2 and S3. Here the phylogeny of the 16S sequences is presented. The first column has the exact sequences, the next six columns present the phylogenetic information, and the final column maps the sequences and the phylogeny to the ASV number used in Data S1.

Data S3. Abundance data at OTU level Related to STAR Methods and Results section “algal impact on taxonomic composition depends on dilution frequency” and Figure 1. Here the counts after grouping by OTUs is presented for all the samples are presented. The rows represent the samples and columns represent the OTUs. The naming convention of the samples is the same as in Data S1.

Data S4. Phylogeny of OTU data Related to STAR Methods and Results section “algal impact on taxonomic composition depends on dilution frequency” and Figure 1. This table represents the taxonomy of the OTUs, including the feature ID and the confidence as given by the algorithm. The final column maps the phylogeny to the OTU number used in Data S1.

Data S5. p values from Fisher exact test of abiotic taxa Related to STAR Methods and Results section “dynamics along PC1 are driven by abiotic factors.” The p values of the taxa contributing to displacement of the communities along PC1 for the analysis with the rare taxa removed, is presented here, according to the analysis described in STAR Methods section “displacement along PC1” for both soil samples. The Bonferroni corrected p value is also mentioned.

Data S6. p values from Fisher exact test of abiotic taxa, without removing the rare taxa Related to STAR Methods and Results section “dynamics along PC1 are driven by abiotic factors.” The p values of the taxa contributing to displacement of the communities along PC1 for the analysis without removing the rare taxa, is presented here, according to the analysis described in STAR Methods section “displacement along PC1” for both soil samples. The Bonferroni corrected p value is also mentioned.

Data S7. GC-MS data of algal spent media Related to STAR Methods and Figure S17. The concentrations of the compounds secreted by *C. reinhardtii* on its own as measured by GC-MS and described in “Chemical analysis of algal exudates” are presented here. The concentrations of these compounds in the media is subtracted from measurement to assay for increasing or decreasing concentrations in time.

Data S8. Sequence, phylogeny, abbreviation, and origin of the bacterial isolates Related to STAR Methods and Results section “algal impact on bacterial isolates varies,” “bacterial isolates inhibit algal growth,” and “bacterial carbon use preferences predict enrichment rates.” For the 21 isolated bacterial strains, the sequence, taxonomy, abbreviation of the names used in Figure 6, and the community from which it was isolated are presented here. Note that for most of these isolates, we obtained multiple clones of the same isolate from different communities. The particular community from which the isolate was used for the experiments are presented here. The abbreviations used in Figure 6 and Figure S17A are included.

Data S9. Phylogenetic tree Related to STAR Methods and Figure S17. The OTU dataset with the rare taxa removed, as described in STAR Methods, is used to create a newick tree file, which was used for Unifrac analysis.
