# Supplementary material for: Algae drive convergent bacterial community assembly at low dilution frequency
Source: iScience. 2023 May 16;26(6):106879. doi: 10.1016/j.isci.2023.106879 (PMC10238937; doi:10.1016/j.isci.2023.106879)
Supplement: Document S1. Figures S1–S19 and Tables S1–S7 [file mmc1.pdf]

iScience, Volume *26*

Supplemental information

Algae drive convergent bacterial community assembly at low dilution frequency

Kaumudi H. Prabhakara and Seppe Kuehn

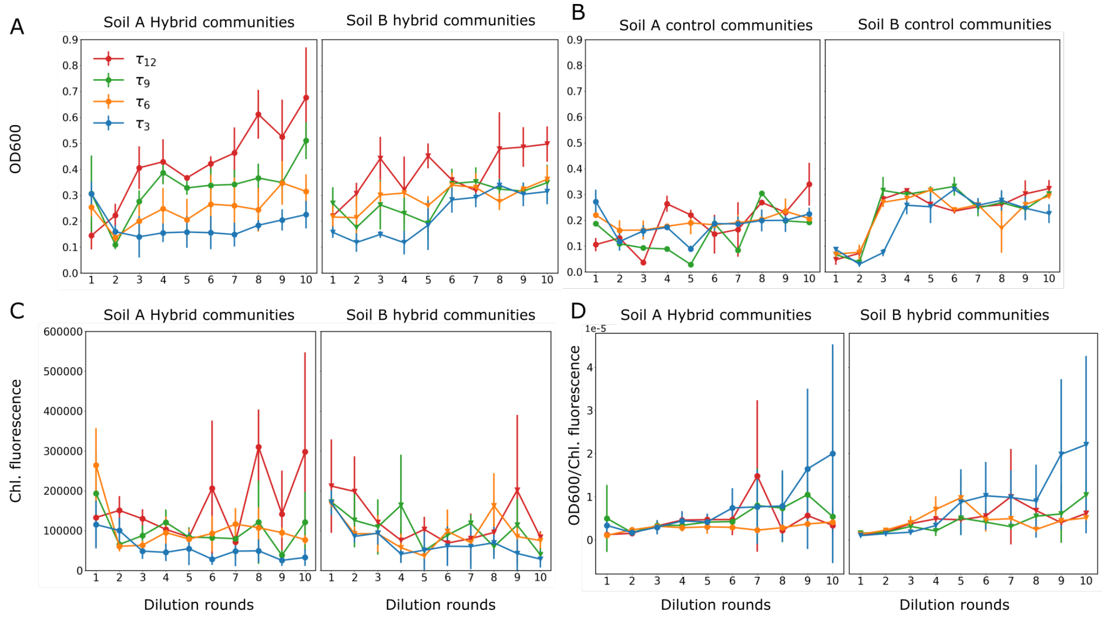

Figure S1: OD600 and Chlorophyll fluorescence measurements. Related to STAR Methods and Results Section 'Algal impact on taxonomic composition depends on dilution frequency' The two columns in each panel correspond to the two soil samples. The error bars represent standard deviation over replicate communities. A shows the changes in OD600 after the end each dilution round for hybrid communities. B shows the changes in OD600 after the end each dilution round for control communities. C shows the changes in the auto-fluorescence of the chlorophyll content in the algae across dilution rounds for the hybrid communities. D shows the ratio of the above two quantities, i.e., OD 600:Chlorophyll content over dilution rounds for the hybrid communities.

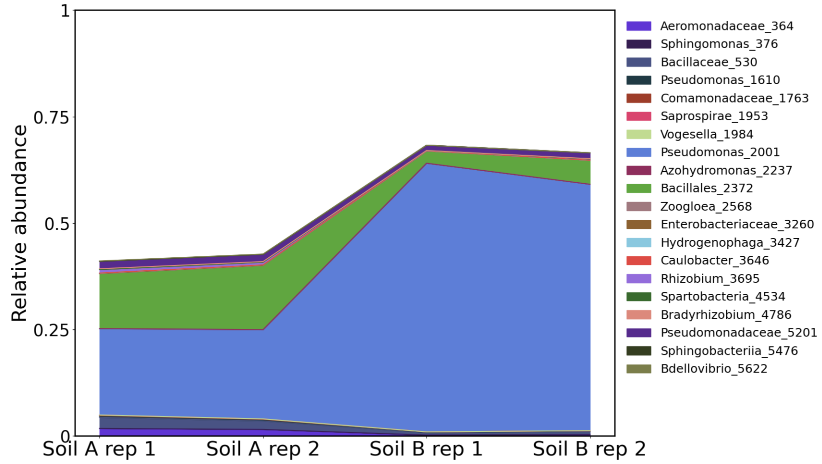

Figure S2: Composition of initial soil inoculates. Related to STAR Methods and Results Section 'Algal impact on taxonomic composition depends on dilution frequency' The taxonomic composition of the initial soil inoculates, (after the treatment in the dark with drugs), with two replicates each, based on 16S sequencing is shown here. Only those OTUs that have a relative abundance greater than 20% at any time in any community are plotted. The relative abundance is plotted on the y-axis. The color map is the same as the color map of Figure 2 in the main text. The labels are the genus name of the OTU followed by the OTU number assigned during analysis. If the genus of a OTU is unknown, the next higher known classifier is used.

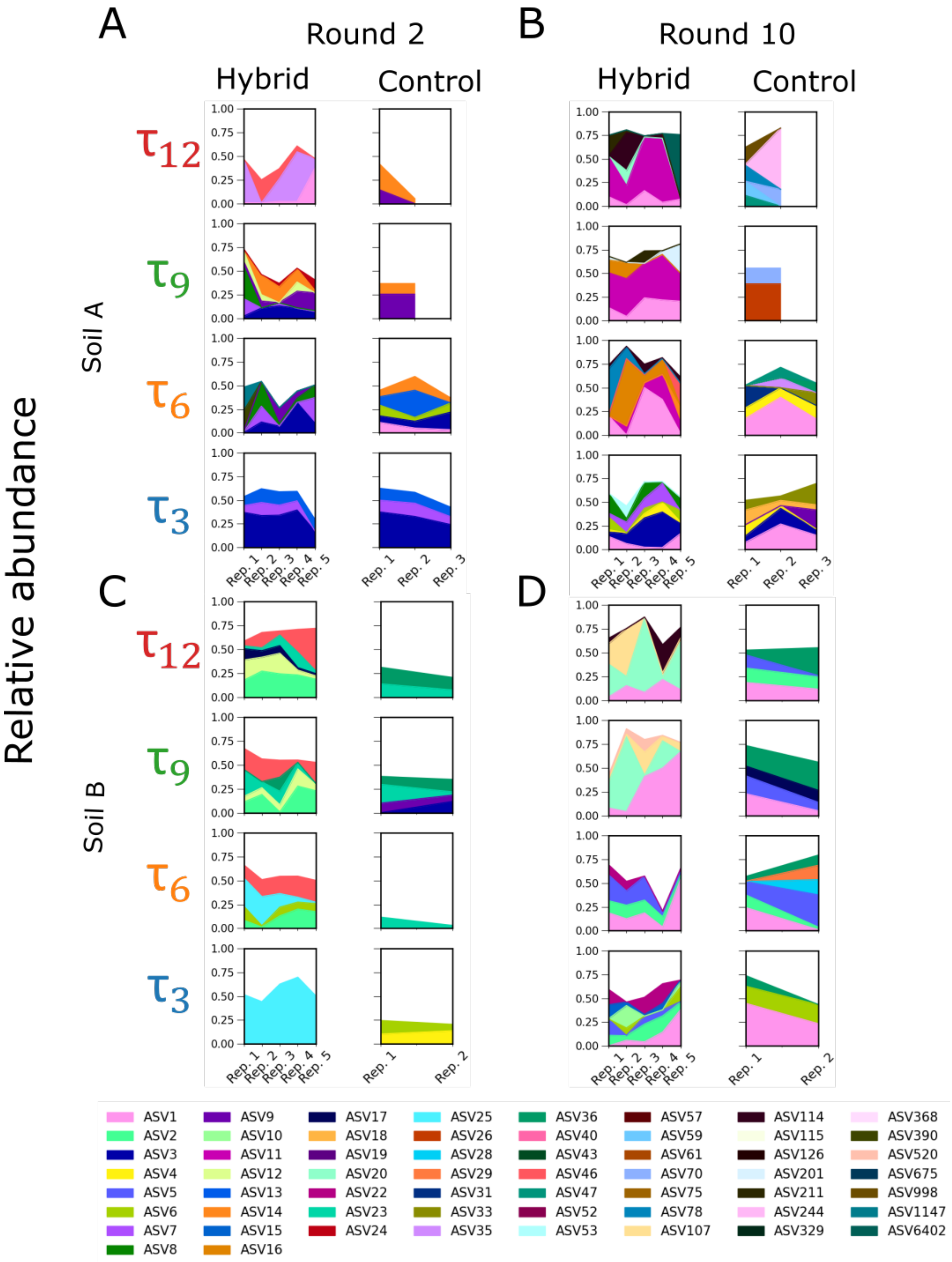

Figure S3: Composition of the communities at the ASV level. Related to STAR Methods and Results Section 'Algal impact on taxonomic composition depends on dilution frequency' Same layout as Figure 2 of the main text. The taxonomic composition of the dominant taxa of the hybrid and control communities at the ASV level for serial dilution rounds 2 and 10 are shown here. By dominant taxa we mean those taxa that occur at a relative abundance of at least 20% in any community at any time point.

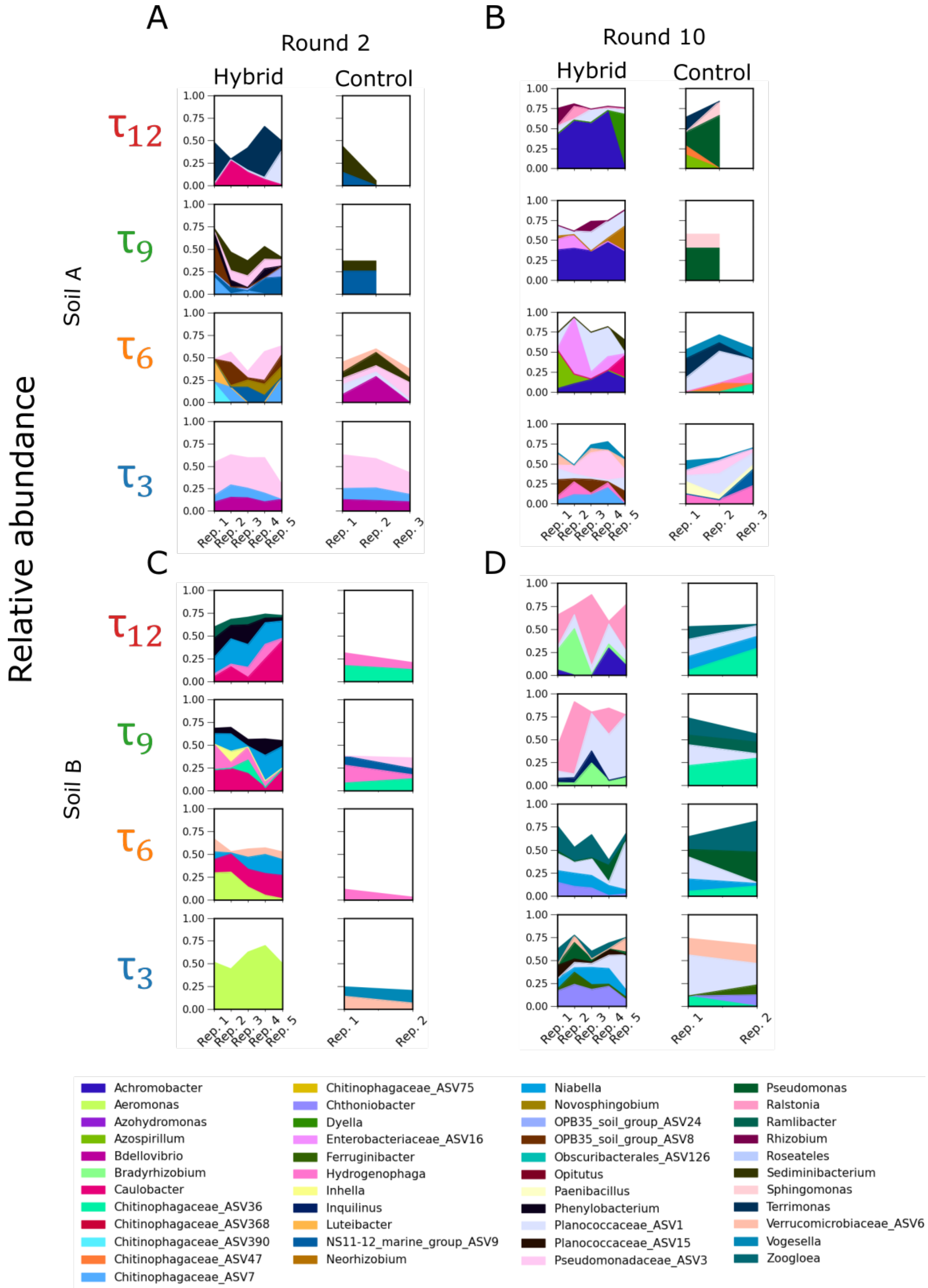

Figure S4: Composition of the communities at the genus level. Related to STAR Methods and Results Section 'Algal impact on taxonomic composition depends on dilution frequency' Same layout as Figure 2 of the main text. The taxonomic composition of the dominant taxa of the hybrid and control communities at the genus level for serial dilution rounds 2 and 10 are shown here. By dominant taxa we mean those taxa that occur at a relative abundance of at least 20% in any community at any time point. Taxa with the same genus are grouped together. If the genus is not assigned, the next taxonomic classificiation is used along with the assigned ASV number.

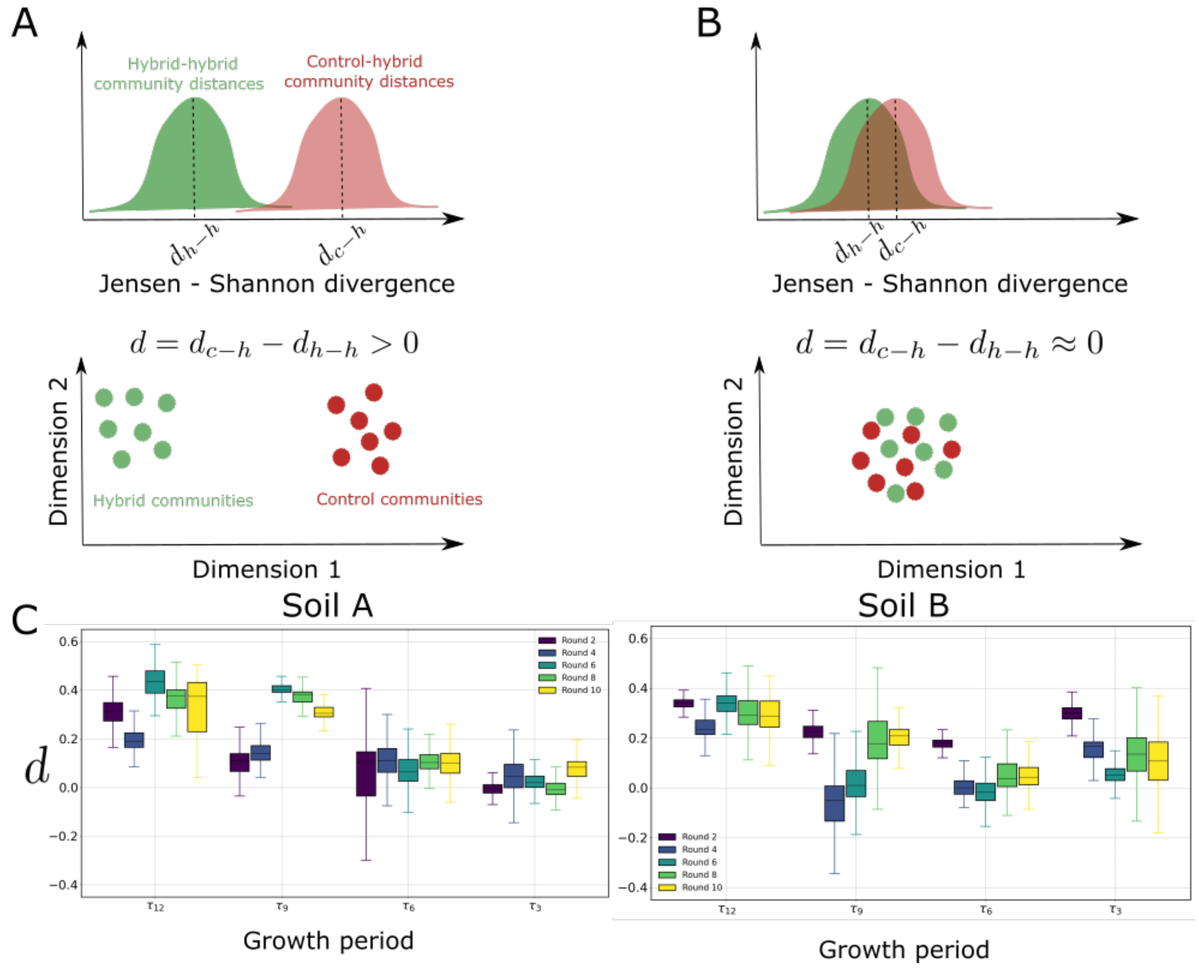

Figure S5: Jensen-Shannon divergence between hybrid and control communities. Related to STAR Methods and Results Section 'Algal impact on taxonomic composition depends on dilution frequency' The Jensen-Shannon divergence (JSD) is computed between all pairs of communities. Then, the for each soil sample, for each dilution round, and for each growth period, the distribution of JSD is plotted for hybrid - hybrid communities and control-hybrid communities. A and B show hypothetic sketches of what these two distributions of JSD might look like. A shows the case where hybrid communities are taxonomically more similar to each other than they are to the control communities because the median distance between hybrid communities (d_{h-h}) is smaller than the median distance between control and hybrid communities (d_{c-h}), and $d = d_{c-h} - d_{h-h}$ is positive. Such a situation could be represented in the bottom row of A. If the distributions are as sketched in B, then the hybrid communities are taxonomically similar to the control communities because the median distance between hybrid communities (d_{h-h}) is similar to the median distance between control and hybrid communities (d_{c-h}), and $d = d_{c-h} - d_{h-h}$ is roughly zero. Such a situation would look like the sketch in the bottom row of B. In C, d is computed across all replicates and plotted on the y-axis for each dilution round and growth period plotted along the x-axis. By dilution round 10, in both soil samples d is significantly positive for the τ_{12} and τ_9 communities (p-values < 0.002, testing for the null hypothesis that d is equal to or less than zero.) However, the τ_3 communities do not have a significantly positive d (p-values 0.13 and 0.07).

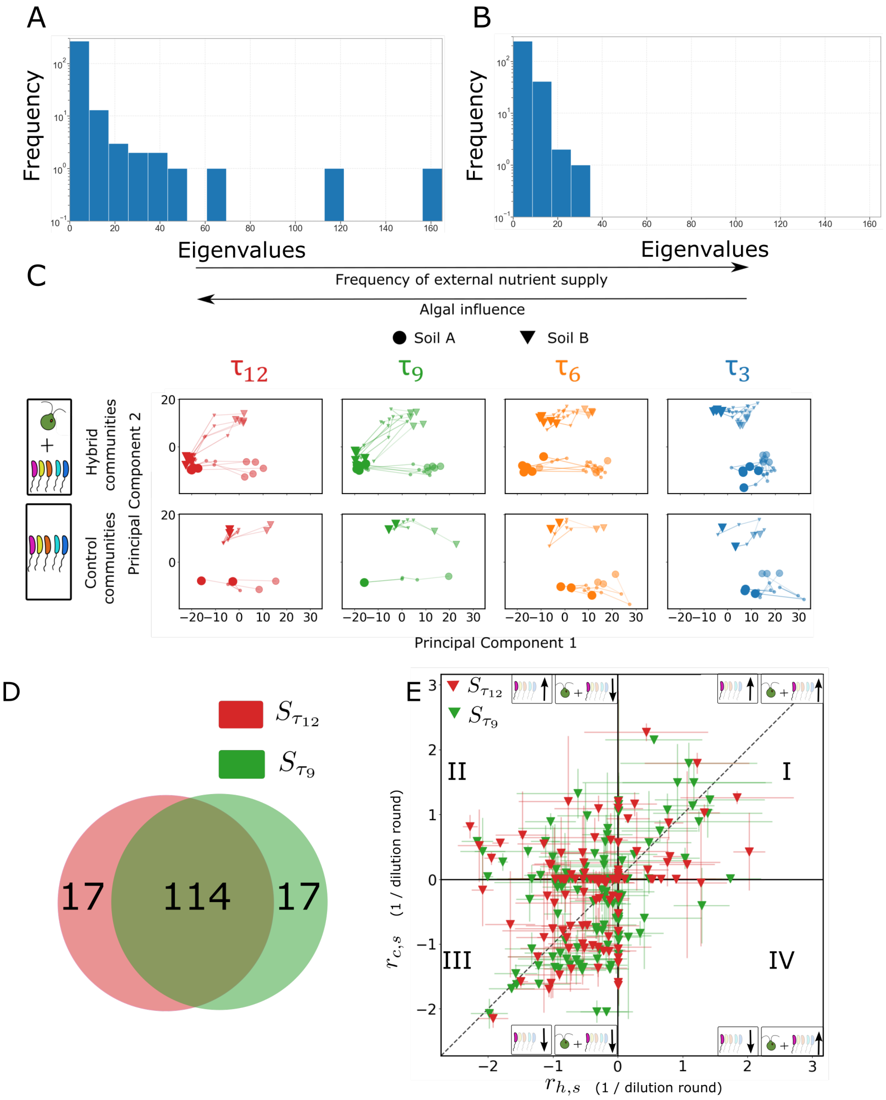

Figure S6: Principle components analysis without removal of rare taxa. Related to STAR Methods and Results Section 'Dimensionality reduction shows algae drive convergence of bacterial community composition' PCA was performed on the entire data set without any removal of rare taxa. A shows the distribution of eigenvalues for the data, showing two modes with high eigenvalues. B shows the distribution of eigenvalues for shuffled data. The two dominant modes are absent. C shows the PCA results on the entire data without removal of rare taxa. The circles and triangles differentiate the two soil samples. The different colors represent the different growth periods. The intermediate translucent markers represent the initial round of serial dilution, the large solid markers represent the final round of serial dilution. The smallest markers represent the intermediate rounds of serial dilution. The results are very similar to Figure 4 in the main text, showing that the two soil samples converge for hybrid τ_9 and and τ_{12} communities, but not for the control or the τ_3 and τ_6 communities. D shows the intersection of the biotic taxa, i.e. those that contribute to the motion along PC2 for τ_{12} ($S_{\tau_{12}}$) and τ_9 (S_{τ_9}) hybrid communities of soil sample B. As with thresholded data (Figure 5C), majority of the taxa that cause displacement along PC2 are common to the two growth periods. E shows the enrichment plot of these biotic taxa. As for the analysis with rare taxa removed, (Figure 5E, main text), most of these taxa are on the left half of the figure.

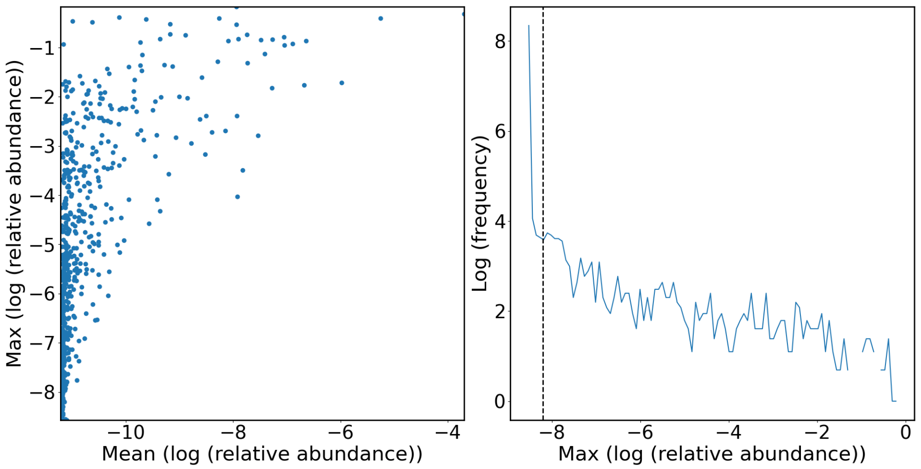

Figure S7: Selecting the threshold for removal of OTUs for PCA analysis. Related to STAR Methods and Results Section 'Dimensionality reduction shows algae drive convergence of bacterial community composition' We computed the maximum and mean of logarithm of the relative abundance of all taxa across all dilution rounds and communities. In the left panel this maximum and mean are plotted for all the taxa. We note that majority of the taxa have a very low mean and maximum relative abundance across all samples and all times, indicating they are rare in all communities at all times. To set a cut-off, we plot the frequency of the maximum of the logarithm of the relative abundance across all dilution rounds and for all samples for each OTU in the right panel. The sharp rise in frequency below -8 shows that, as expected, many taxa have a low maximum relative abundance across all time points and samples. Based on this curve, the cutoff is set at a maximum logarithm of relative abundance of -8.2 indicated by the vertical dashed line in the right panel. Out of 5236 OTUs, this process removed 4360 OTUs.

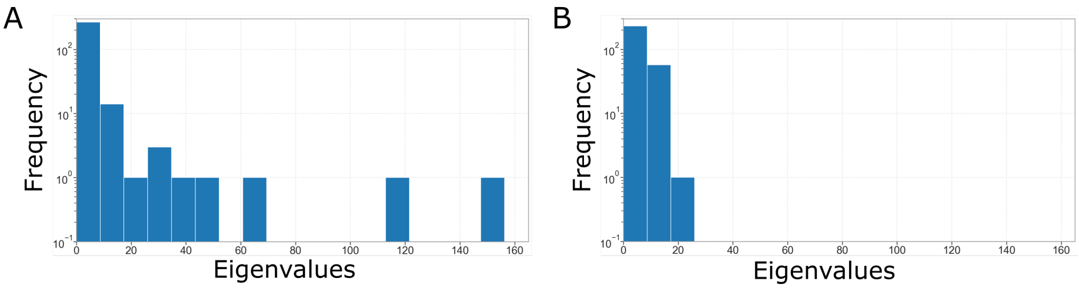

Figure S8: Eigenvalue distributions after removing rare taxa. Related to STAR Methods and Results Section 'Dimensionality reduction shows algae drive convergence of bacterial community composition' PCA is performed on the data with the rare OTUs remove, and the frequency of the eigenvalues are plotted in A. The distribution shows that there are two modes with high eigenvalues that are distinct from the rest. Panel B shows the frequency distribution of the eigenvalues for the shuffled data. Here the two modes with the highest eigenvalues are absent.

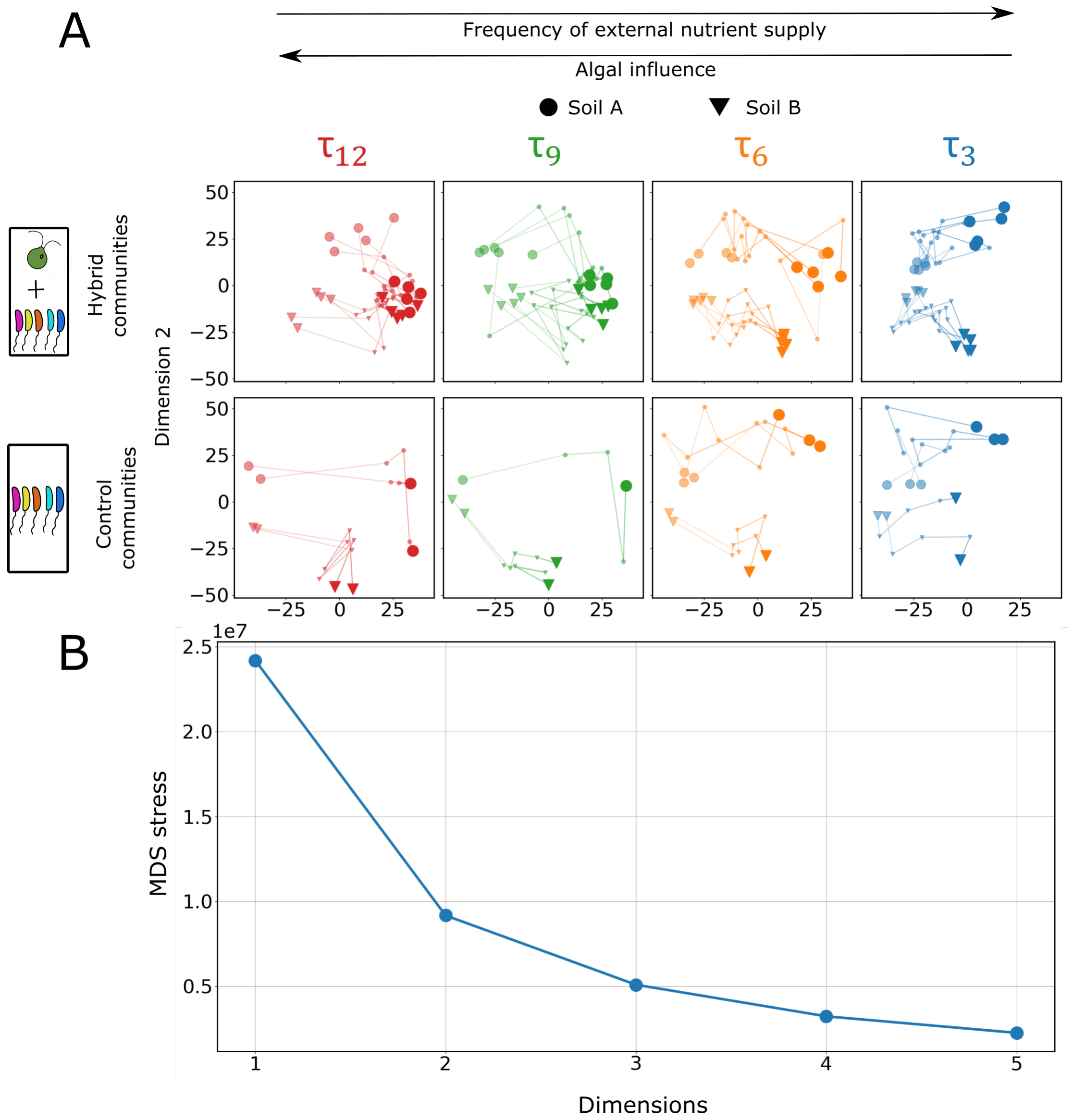

Figure S9: MDS embedding of Aitchison's distances. Related to STAR Methods and Results Section 'Dimensionality reduction shows algae drive convergence of bacterial community composition' Aitchison's distances were computed using the entire data set between all pairs of communities. These distances were then embedded using the metric Multi Dimensional Scaling (MDS, using the MDS function scikit-learn's manifold class) using Euclidean distances in two dimensions, shown in A. The circles and triangles differentiate the two soil samples. The different colors represent the different growth periods. The intermediate translucent markers represent the initial round of serial dilution, the large solid markers represent the final round of serial dilution. The smallest markers represent the intermediate rounds of serial dilution. As with the PCA analysis (Figure 4), we observe that the τ_{12} and τ_9 hybrid communities converge by dilution round 10, whereas the τ_3 and τ_6 hybrid communities and none of the control communities converge. B shows the stress of the MDS embedding.

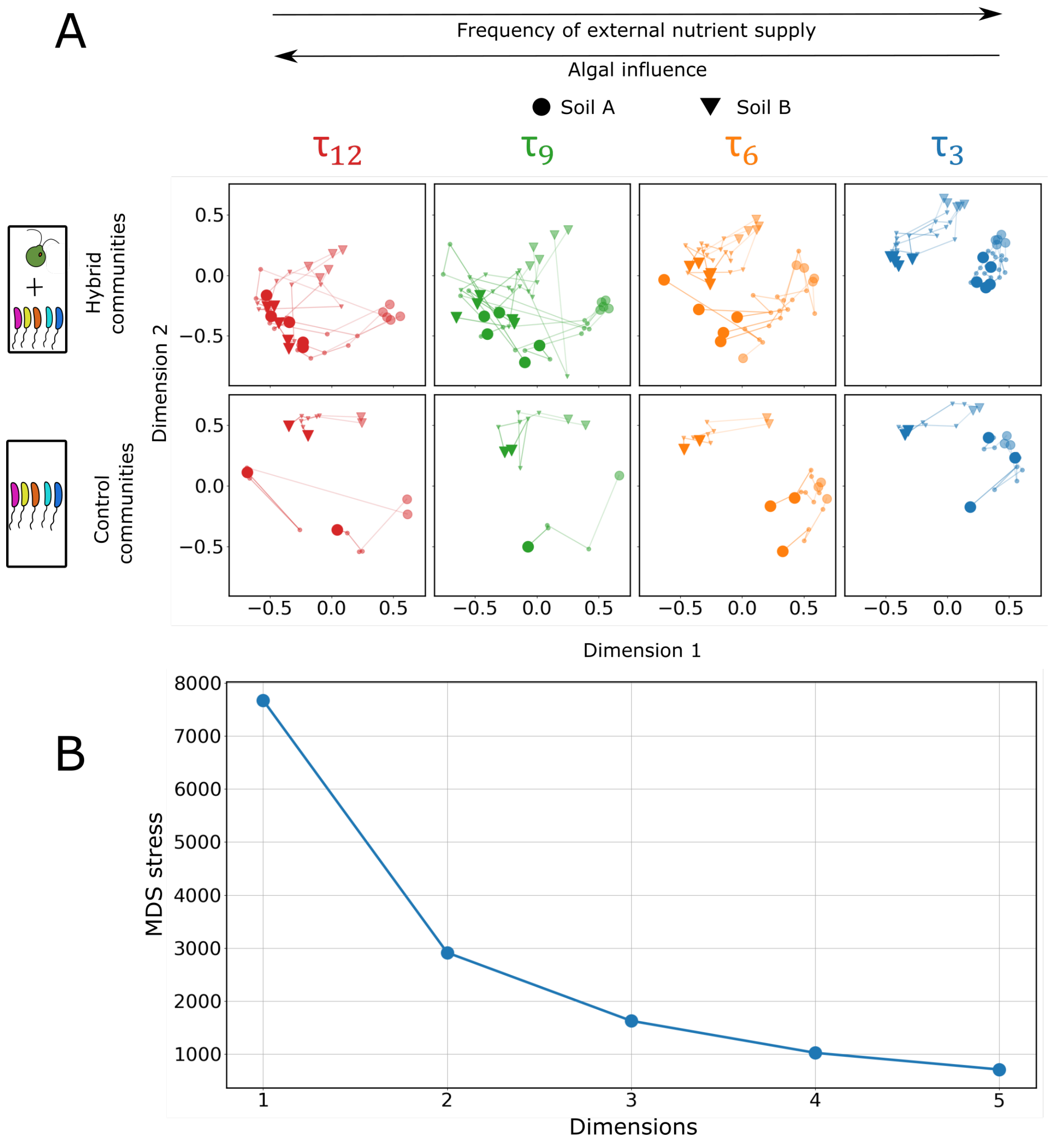

Figure S10: MDS embedding of the Unifrac distances. Related to STAR Methods and Results Section 'Dimensionality reduction shows algae drive convergence of bacterial community composition' Unifrac distances were computed on the data set with rare OTUs removed using a phylogenetic tree, between all pairs of communities. These distances were then embedded using the metric Multi Dimensional Scaling (MDS, using the MDS function scikit-learn's manifold class) using Euclidean distances in two dimensions, shown in A. The circles and triangles differentiate the two soil samples. The different colors represent the different growth periods. The intermediate translucent markers represent the initial round of serial dilution, the large solid markers represent the final round of serial dilution. The smallest markers represent the intermediate rounds of serial dilution. As with the PCA analysis (Figure 4, main text), we observe that the τ_{12} and τ_9 hybrid communities converge by dilution round 10, whereas the τ_3 and τ_6 hybrid communities and none of the control communities converge. B shows the stress of the MDS embedding.

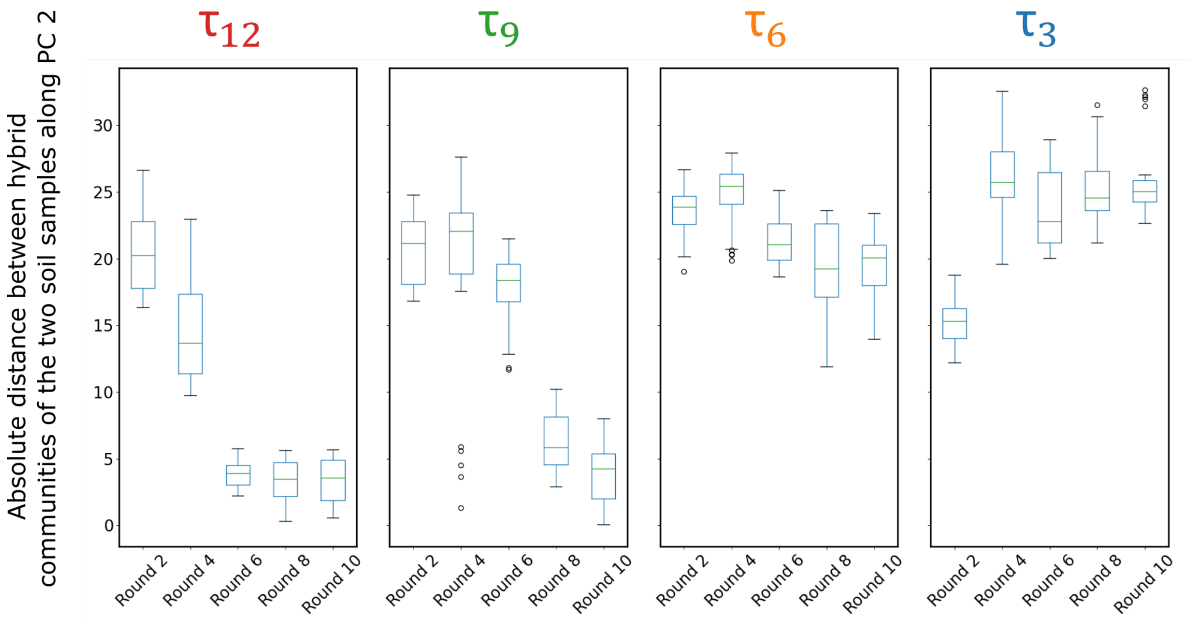

Figure S11: Distance between τ_3 hybrid communities of the two soil samples does not decrease. Related to STAR Methods and Results Section 'Convergence along principal component 2 arises from inhibition of bacteria in the presence of algae' The absolute distance between the hybrid communities along PC2 are plotted for the four growth periods. For τ_{12} and τ_9 communities, the distance decreases, signifying convergence, while for τ_3 communities, the distance increases over dilution rounds.

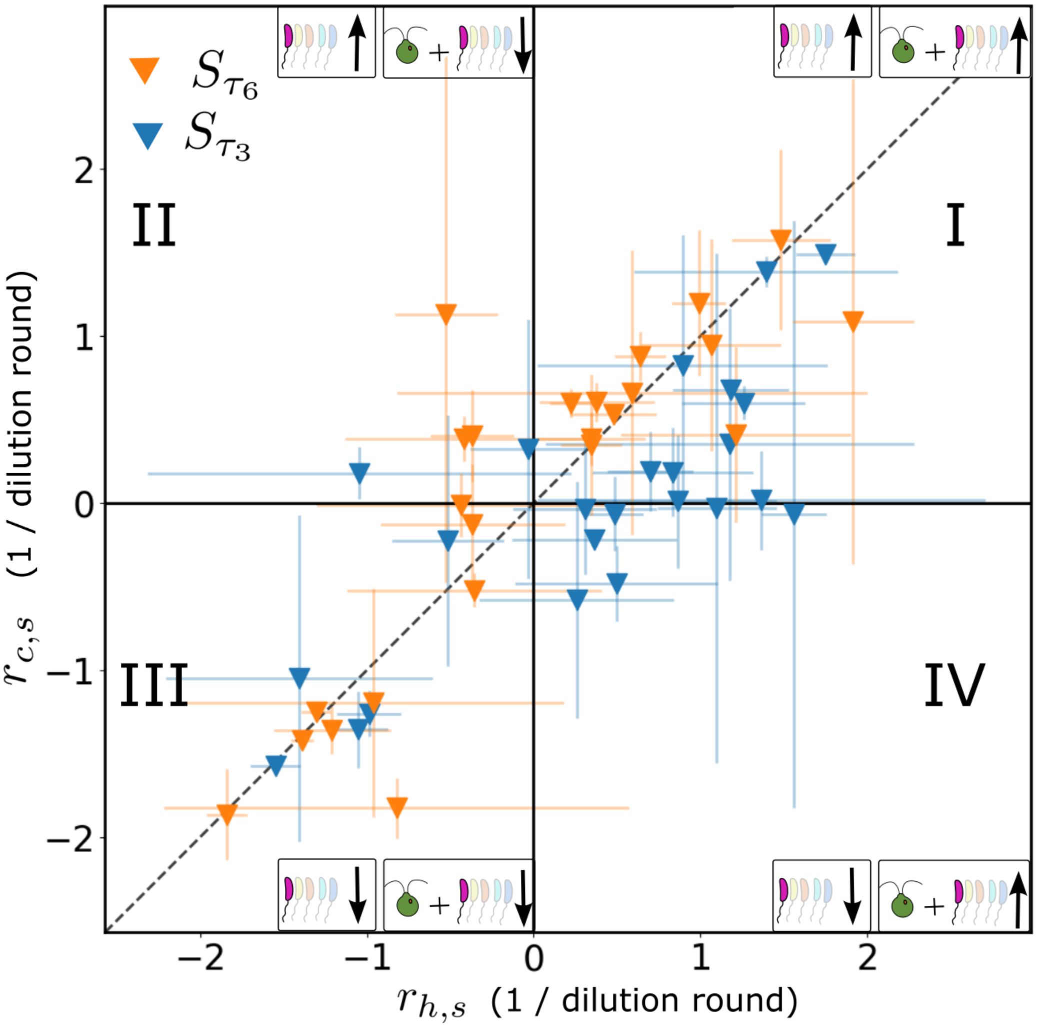

Figure S12: Enrichment plot for biotic taxa in τ_6 and τ_3 hybrid communities. Related to STAR Methods and Results Section 'Convergence along principal component 2 arises from inhibition of bacteria in the presence of algae' The construction of this plot is identical to Figure 5D of the main text. For the biotic taxa selected for their contribution to movement along PC2 in the τ_{12} and τ_9 hybrid communities of soil sample B, their enrichment in the τ_6 and τ_3 hybrid communities are computed as described in the main text and plotted here. As opposed to the τ_9 and τ_{12} hybrid communities (Figure 5D), in the τ_3 and τ_6 hybrid communities, these taxa dominantly lie on the diagonal, where the presence of *C. reinhardtii* has no significant effect on the enrichment dynamics.

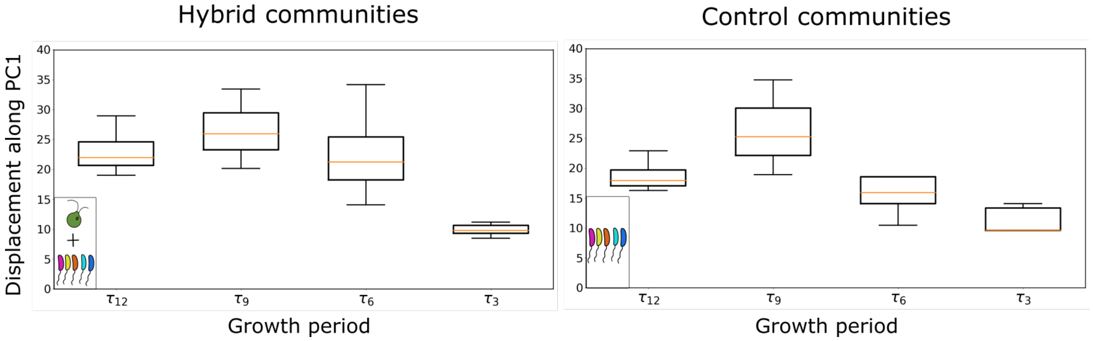

Figure S13: Displacement along PC1 depends on the growth period. Related to STAR Methods and Results Section 'Dynamics along PC1 are driven by abiotic factors' Exogenous nutrient supply rate, which is equivalent to the growth period and is the experimentally controlled abiotic factor, controls the displacement along PC1, the abiotic axis. Communities for which the exogenous nutrients are supplied very frequently, move shorter distances along PC1 compared to the communities where the exogenous nutrient supply is infrequent. This is true for both hybrid (left panel) and control (right panel) communities, for both soil samples. With the null hypothesis that the distribution of displacements laong PC1 is the same for the τ_{12} and τ_3 communities, the Kolmogorov Smirnoff test gives a p-value of 2×10^{-8}, indicating that the null hypothesis is false.

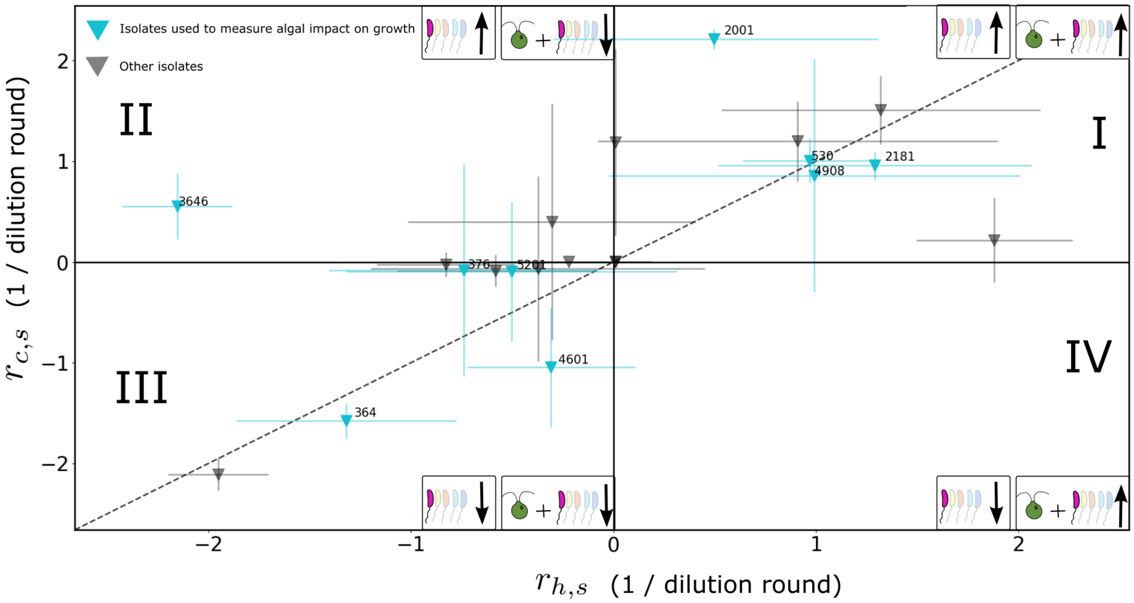

Figure S14: Enrichment plot for all isolates. Related to STAR Methods and Results Section 'Algal impact on bacterial isolates varies' For the 21 isolated bacterial strains, their enrichment in the control and hybrid communities, averaged over the τ_9 and τ_{12} growth periods are plotted. The error bars indicate standard deviation. The isolates span all four quadrants of the plot. The isolates in blue are used in the measurement of algal impact on growth in Figure 6D. For visual clarity, only their assigned OTU numbers are shown.

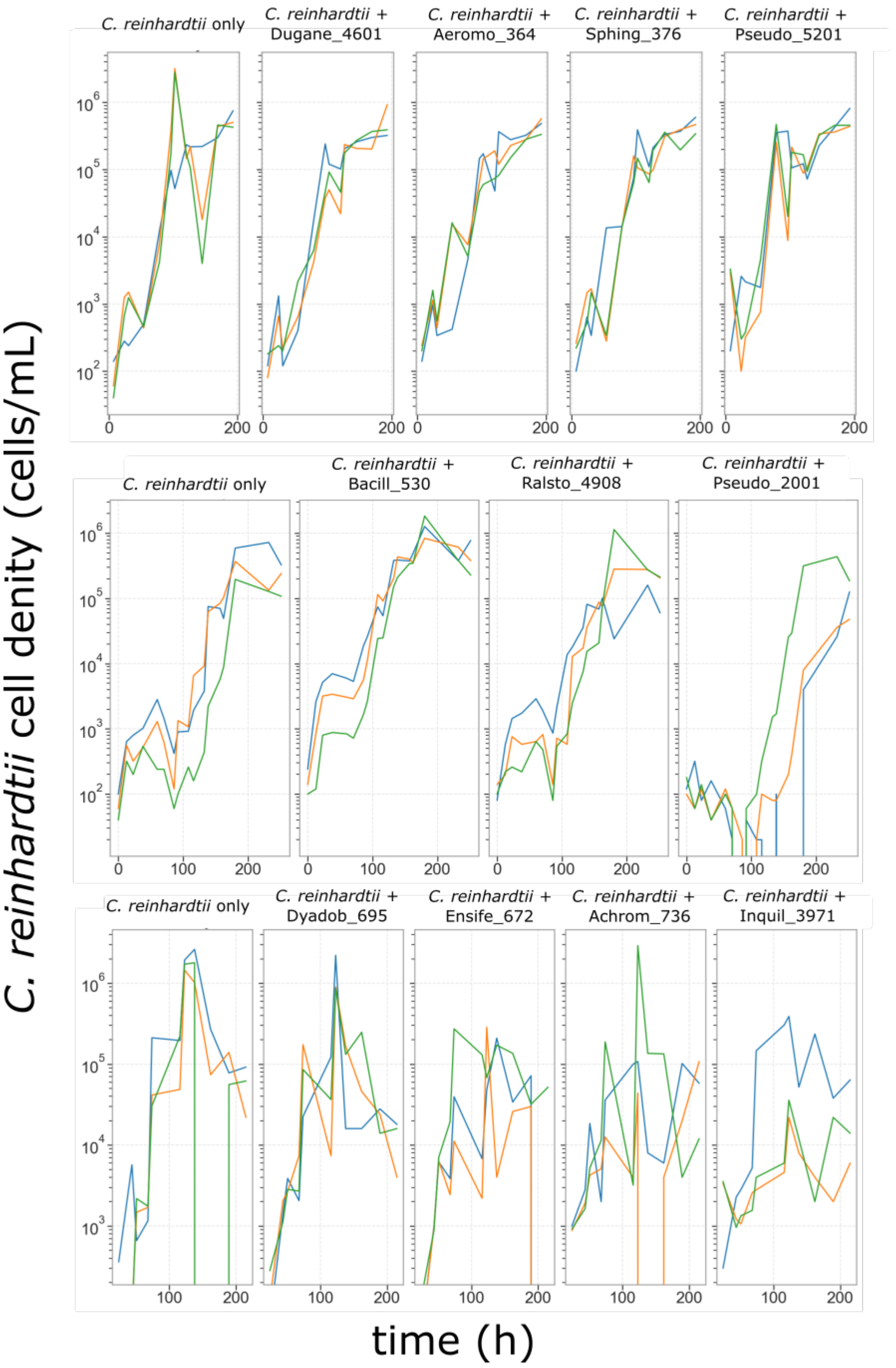

Figure S15: Raw data from flow cytometry. Related to STAR Methods and Results Section 'Bacterial isolates inhibit algal growth' The raw data from flow cytometry is plotted here. There were 3 runs in total, represented by each row in the figure. For each run, we had the control of *C. reinhardtii* growing on their own. There were three replicates for each measurement, represented by the three curves in each panel.

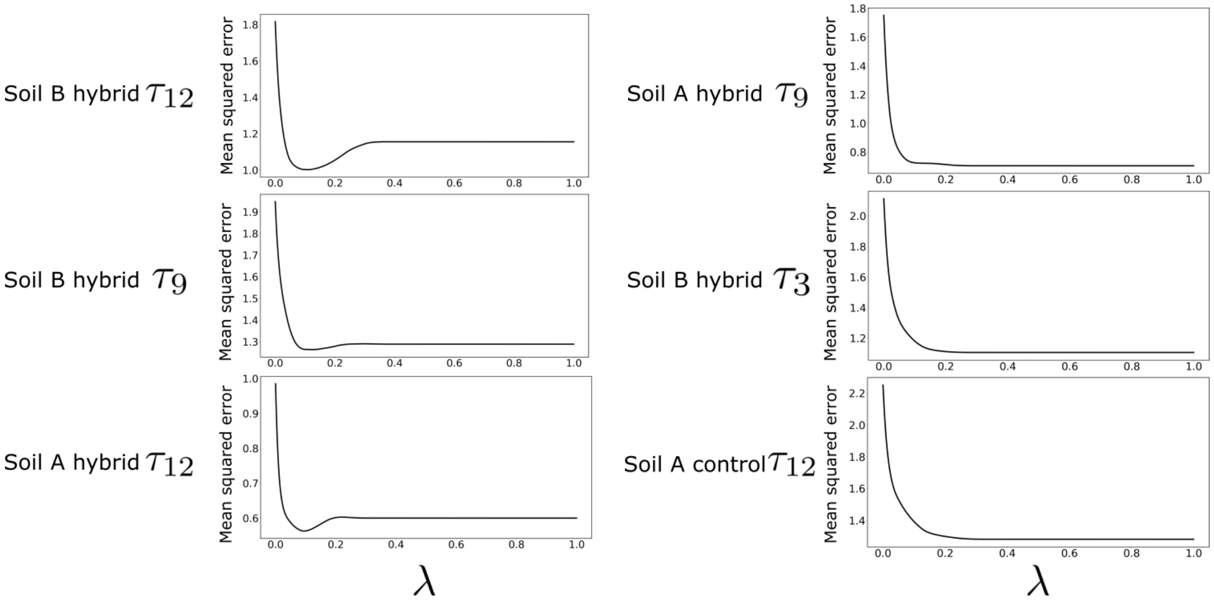

Figure S16: Determination of optimal hyperparameter (λ) for LASSO regression via cross-validation. Related to STAR Methods and Results Section 'Bacterial carbon use preferences predict enrichment rates' Mean squared error in held out data determined by iterated 4-fold cross validation for a range of λ. Here, in the first column, we show the cases where the LASSO regression finds an optimal penalty coefficient λ: there is a well defined minimum in the mean squared error for a particular λ. In the second column, we show that the LASSO regression fails to find a minimum for λ, because there is no well defined minimum for the mean squared error for a range of λ. Therefore the regression fails in these cases, and as shown in[1], in such cases, the LASSO regression and only returns an intercept and there is likely no sparse solution to the regression problem.

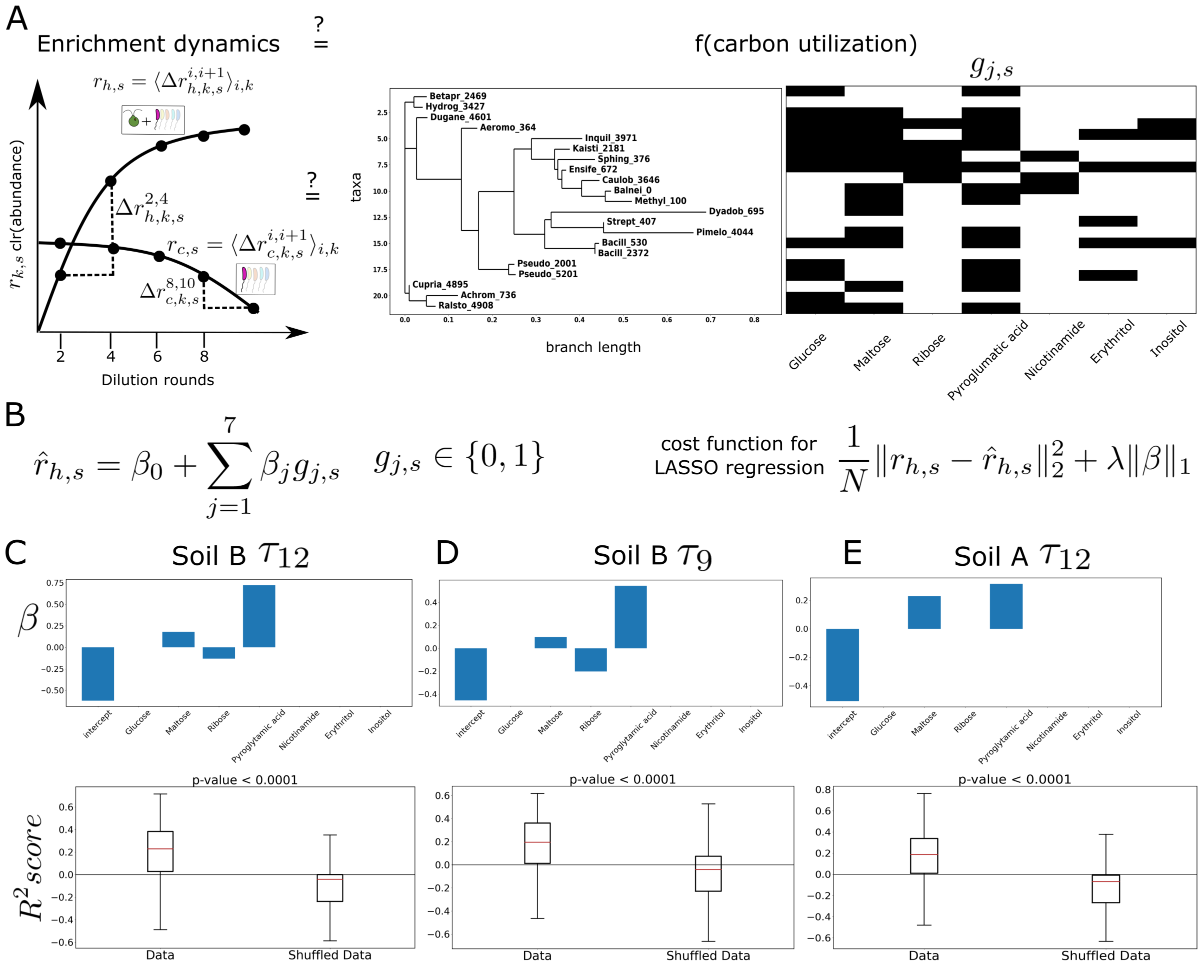

A
Enrichment dynamics
f(carbon utilization)
Dilution rounds
branch length
taxa
Glucose
Maltose
Ribose
Pyroglumatic acid
Nicotinamide
Erythritol
Inositol
B
cost function for
LASSO regression
C
Soil B
D
Soil B
E
Soil A
p-value < 0.0001
Data
Shuffled Data

Figure S17 ***(preceding page)*****: Carbon catabolism weakly predicts enrichment rates in long growth period hybrid communities. Related to STAR Methods and Results Section 'Bacterial carbon use preferences predict enrichment rates'** A. We ask if the enrichment rates ($r_{h,s}$, $r_{c,s}$) of the bacterial isolated strains are related to their ability to grow on a carbon source, represented here as a binary matrix $g_{j,s}$ for the isolated strains s and carbon source j. The isolated strains are ordered according to their position on a phylogenetic tree. The naming convention is first six letters of their genus followed by the assigned number. If the genus is not assigned, the next assigned taxonomy is used. The binary matrix to the right indicates growth or no growth on each carbon source as measured in a plate reader experiment (see Methods). B. The enrichment rate of the isolate s is predicted ($\hat{r}_{h,s}$) through a regression against its carbon consumption ability $g_{j,s} \in 0, 1$, with coefficients β_j and intercept β_0. Regularized, LASSO regression is performed, with the cost function as shown, where N is the number of samples (here, 21 isolates), $r_{h,s}$ the true enrichment, $\hat{r}_{h,s}$ the predicted enrichment, λ is the penalty coefficient and β is the vector of the regression coefficients β_j and β_0. The optimal value of λ is found first by iterated cross-validation with a 4 fold split and 100 repeats, and then the regression coefficients are determined at the optimal hyperparameter $\hat{\lambda}$ (see Methods and Figure S16) C. The top panel shows the regression coefficients β_j and the intercept β_0 for enrichment rates for isolates in Soil B τ_{12} hybrid communities. The LASSO regression picks Maltose, Ribose and Pyroglutamic acid as the carbon sources with non-zero regression coefficients. To test if the regression has out of sample predictive power, we train the regression on part of the data at the optimal hyperparameter $\hat{\lambda}$, and test its prediction on the rest. We report an R^2 on the held out data to measure the out-of-sample predictive power. The bottom panel shows the distribution of this R^2 score on the data and on the data obtained by shuffling the enrichment rates. A bootstrap test is performed with the null hypothesis that the median of the R^2 score of the shuffled data is larger than or equal to that of the actual data. The low p-value suggests that this null is false, and that the median of the R^2 distribution of the actual data is higher than that of the shuffled data, indicating that the LASSO regression is significantly predictive on the data. D. The top panel shows the regression coefficients β_j and the intercept β_0 for Soil B τ_9 hybrid communities. The regression picks Maltose, Ribose and Pyroglutamic acid as the carbon sources with the predictive power. The distribution at the bottom panel of the R^2 scores for the data and the shuffled data shows that LASSO regression has a significant predictive power on the data. E. The top panel shows the regression coefficients β_j and the intercept β_0 for Soil A τ_{12} hybrid communities. The LASSO regression picks Maltose and Pyroglutamic acid as the carbon sources with the predictive power. The distribution at the bottom panel of the R^2 scores for the data and the shuffled data shows that the regression has significant out-of-sample predictive power.

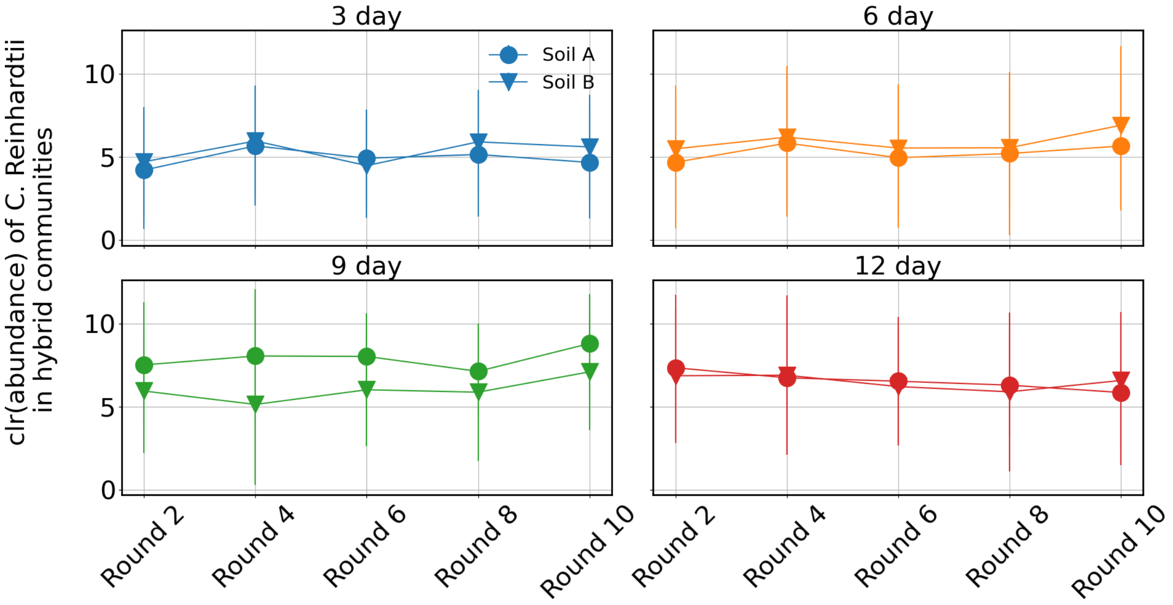

Figure S18: Estimation of *C. reinhardtii* content from sequence data. Related to Results Section 'Algal impact on taxonomic composition depends on dilution frequency' and Figure 2 The relative abundance of all OTUs was calculated, and then the center-log-transform was taken as described in the Methods. For each hybrid community of a given soil sample, growth period and dilution round, the average of the clr transformed abundance corresponding to the chloroplast reads, defined as the dominant OTU in the sample containing only *C. reinhardtii*, was computed across the five replicates. This average is plotted here with the standard deviation as the error bar. The communities with 12 day and 9 day growth periods have slightly higher chloroplast reads compared to the communities with the 3 day growth period. This is consistent with the chlorophyll measurements.

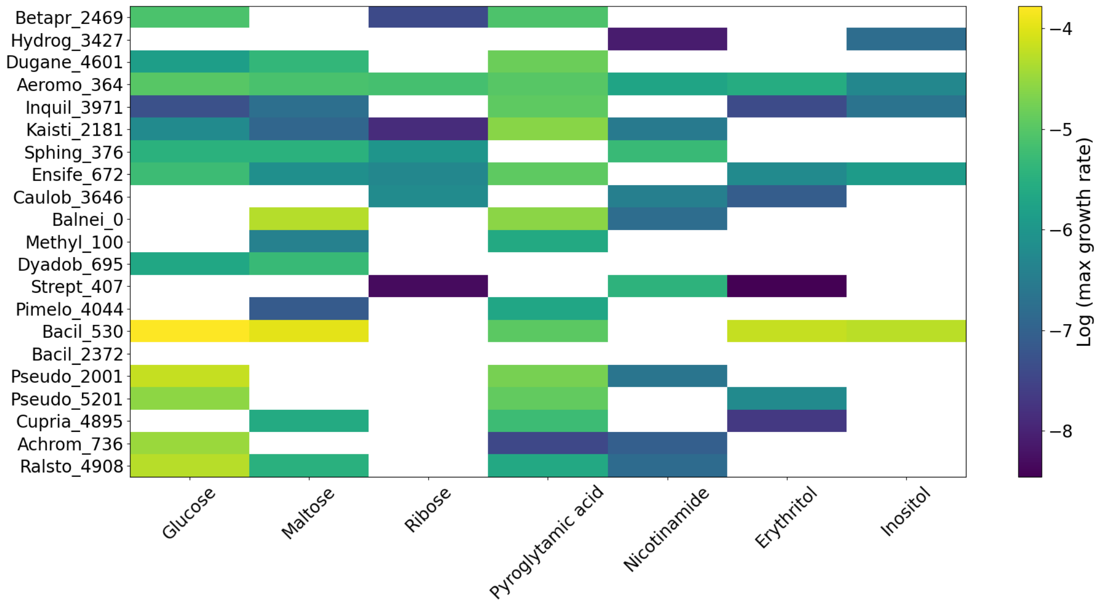

Figure S19: Growth rates of the isolates on different carbon sources. Related to STAR Methods and Results Section 'Bacterial carbon use preferences predict enrichment rates' The growth of the 21 isolates on 7 carbon sources was monitored on plate readers with three replicates for each isolates in each carbon source. The maximum growth rate was computed by fitting a spline to the growth curves and finding the maximum of the first derivative. The logarithm of these maximal growth rates are plotted here. The white color at certain carbon sources indicates that the isolates did not grow on those carbon sources.

Compound	Concentration
$C_6H_{12}O_6$ (glucose)	1.333 mM
NH_4Cl	8 mM
KH_2PO_4, K_2HPO_4	3.1 mM
$MgSO_4$	0.1 mM
$CaCl_2$	1 mM
$C_{10}H_{16}N_2O_8$ (EDTA)	5.5 μM
$FeSO_4$	5.5 μM
H_3BO_4	15 μM
$ZnSO_4$	0.5 μM
$MnCl_2$	3.5 μM
Na_2MoO_4	0.58 μM
$CuSO_4$	0.15 μM
$Co(NO_3)_2$	0.8 μM
NaOH	999 μM
$FeSO_4 \cdot 7H_2O$	999 μM
NaCl	999 μM

Table S1: Modified 1/2x Taub medium composition. Related to STAR Methods.

Compound	Concentration
Tris-Cl	20 mM
Sodium EDTA	2 mM
Triton X-100	1.2%
Lysozyme from chicken egg	20 mg/mL

Table S2: Lysis buffer composition for DNA extraction. Related to STAR Methods.

Reagent	Volume
PCR grade water	13 μL
Forward primer (10 μM)	0.5 μL
Reverse primer (10 μM)	0.5 μL
Template DNA	1 μL
Platinum Hot Start Master Mix (2X)	10 μL

Table S3: Reagents for PCR for EMP protocol. Related to STAR Methods.

Temperature	Time	Repeat
94 C	3 min	
94 C	45 s	45x
50 C	60 s	45x
72 C	90 s	45x
72 C	10 min	
4 C	hold	

Table S4: Thermocycler settings for EMP protocol. Related to STAR Methods.

Reagent	Volume
PCR grade water	7 μL
Forward primer (10 μM)	1 μL
Reverse primer (10 μM)	1 μL
Cell suspension	1 μL
Platinum Hot Start Master Mix (2X)	10 μL

Table S5: Reagents for colony PCR. Related to STAR Methods.

Temperature	Time	Repeat
98 C	3 min	
98 C	10 s	40x
55 C	30 s	40x
72 C	45 s	40x
72 C	10 min	
4 C	hold	

Table S6: Thermocycler settings for colony PCR. Related to STAR Methods.

Compound excreted by *C. reinhardtii* in significant quantities
2-O-Glycerol-α-d-galactopyranoside
Digalactosylglycerol
Erythritol
Galacotse
Glyceric acid
Inositol,myo
Malic acid
Maltose
Nicotinamide
Proline
Putrescine
Pyroglutamic acid
Ribose
Threitol
Threonic acid

Table S7: Compounds excreted in significant amounts by *C. reinhardtii* grown on its own as measured by GC-MS. See Methods section "Chemical analysis of algal exudates" and Dataset 7 for more details. Related to STAR Methods.

References

[1] Fraebel, D. T., Gowda, K., Mani, M. & Kuehn, S. Evolution of Generalists by Phenotypic Plasticity. *iScience* **23**, 101678 (2020). URL https://doi.org/10.1016/j.isci.2020.101678.
